# Anticancer potential of rhizome extract and a labdane diterpenoid from *Curcuma mutabilis* plant endemic to Western Ghats of India

**DOI:** 10.1038/s41598-020-79414-8

**Published:** 2021-01-12

**Authors:** T. Soumya, T. Lakshmipriya, Karel D. Klika, P. R. Jayasree, P. R. Manish Kumar

**Affiliations:** 1 Department of Biotechnology, University of Calicut, Malappuram, 673635 Kerala India; 2 Molecular Structure Analysis, German Cancer Research Center (DKFZ), Heidelberg, Germany; 3 School of Health Sciences, University of Calicut, Malappuram, 673635 Kerala India

**Keywords:** Cancer, Cell biology, Drug discovery, Molecular biology, Plant sciences

## Abstract

Zingiberaceae plants are well known for their use in ethnomedicine. *Curcuma mutabilis* Škorničk., M. Sabu & Prasanthk., is an endemic Zingiberaceae species from Western Ghats of Kerala, India. Here, we report for the first time, the anticancer potential of petroleum ether extract from *C. mutabilis* rhizome (CMRP) and a novel labdane diterpenoid, (*E*)-14, 15-epoxylabda-8(17), 12-dien-16-al (Cm epoxide) isolated from it. CMRP was found to be a mixture of potent bioactive compounds including Cm epoxide. Both the extract and the compound displayed superior antiproliferative activity against several human cancer cell lines, without any display of cytotoxicity towards normal human cells such as peripheral blood derived lymphocytes and erythrocytes. CMRP treatment resulted in phosphatidylserine externalization, increase in the levels of intracellular ROS, Ca^2+^, loss of mitochondrial membrane potential as well as fragmentation of genomic DNA. Analyses of transcript profiling and immunostained western blots of extract-treated cancer cells confirmed induction of apoptosis by both intrinsic and extrinsic pathways. The purified compound, Cm epoxide, was also found to induce apoptosis in many human cancer cell types tested. Both CMRP and the Cm epoxide were found to be pharmacologically safe in terms of acute toxicity assessment using Swiss albino mice model. Further, molecular docking interactions of Cm epoxide with selected proteins involved in cell survival and death were also indicative of its druggability. Overall, our findings reveal that the endemic *C. mutabilis* rhizome extract and the compound Cm epoxide isolated from it are potential candidates for development of future cancer chemotherapeutics.

## Introduction

Three quarters of the prescribed anticancer drugs are plant-derived^1^. Classical examples include vinblastine, vincristine, taxol, camptothecin and podophyllotoxin^2,3^. However, only about 5–15% of the 250,000 higher plants have been screened for potential drugs^4^. Despite considerable efforts, clinical use of plant-based, semi-synthetic or synthetic chemotherapeutic agents have been found to be largely inadequate in fulfilling expectations of a safe and affordable cancer cure. Consequently, a constant demand for novel, efficacious and biocompatible anticancer pharmaceuticals bereft of undesirable side-effects, continues to exist^5^. In this context, it is relevant and logical to exploit the extensive biodiversity of unexplored plants in a tropical country like India. The Western Ghats of India represent one of the world’s ten biodiversity hotspots treasuring more than 700 medicinal plants^6,7^.

The ethnomedicinally important family of Zingiberaceae is credited with the isolation of an impressive array of pure compounds with proven anticancer activity. A few notable examples are curcumin/curcuminoids (*Curcuma longa*), zerumbone (*Zingiber zerumbet*), gingerol and shogaol (*Zingiber officinale*) amongst many others^8–13^. *Curcuma mutabilis* Skornick., M. Sabu & Prasanthk. is an unexplored, endemic Zingiberaceae species reported from Western Ghats of Kerala in India^14^. Essential oil isolated from the rhizome of *C. mutabilis*, with estrone methyl ether (3-Methoxyestra-1,3,5(10)-trien-17-one) as the major constituent, was found to possess potent antiproliferative activity against human cancer cell lines, K562 and HCT116^15^.

The present study aimed to evaluate, for the first time, the cytotoxic and antiproliferative potential of different organic solvent extracts, prepared from *C. mutabilis* rhizome using petroleum ether, chloroform, acetone and methanol, against several human cancer cell types. On an initial screening of these, petroleum ether extract (CMRP) was found to possess the most potent activity, which was then investigated further to study its cellular and molecular effects in detail. Subsequent activity-guided isolation of the extract led to the characterization and identification of a labdane diterpenoid, (*E*)-14,15-epoxylabda-8(17),12-dien-16-al (IUPAC) designated as ‘*Curcuma mutabilis* epoxide’ by us. An assessment of the in vitro anticancer potential of this compound in comparison to the parent extract, CMRP, was also carried out.

## Results

### *Curcuma mutabilis* rhizome extracts induce cytotoxicity in a dose-dependent manner

**Figure 1:**
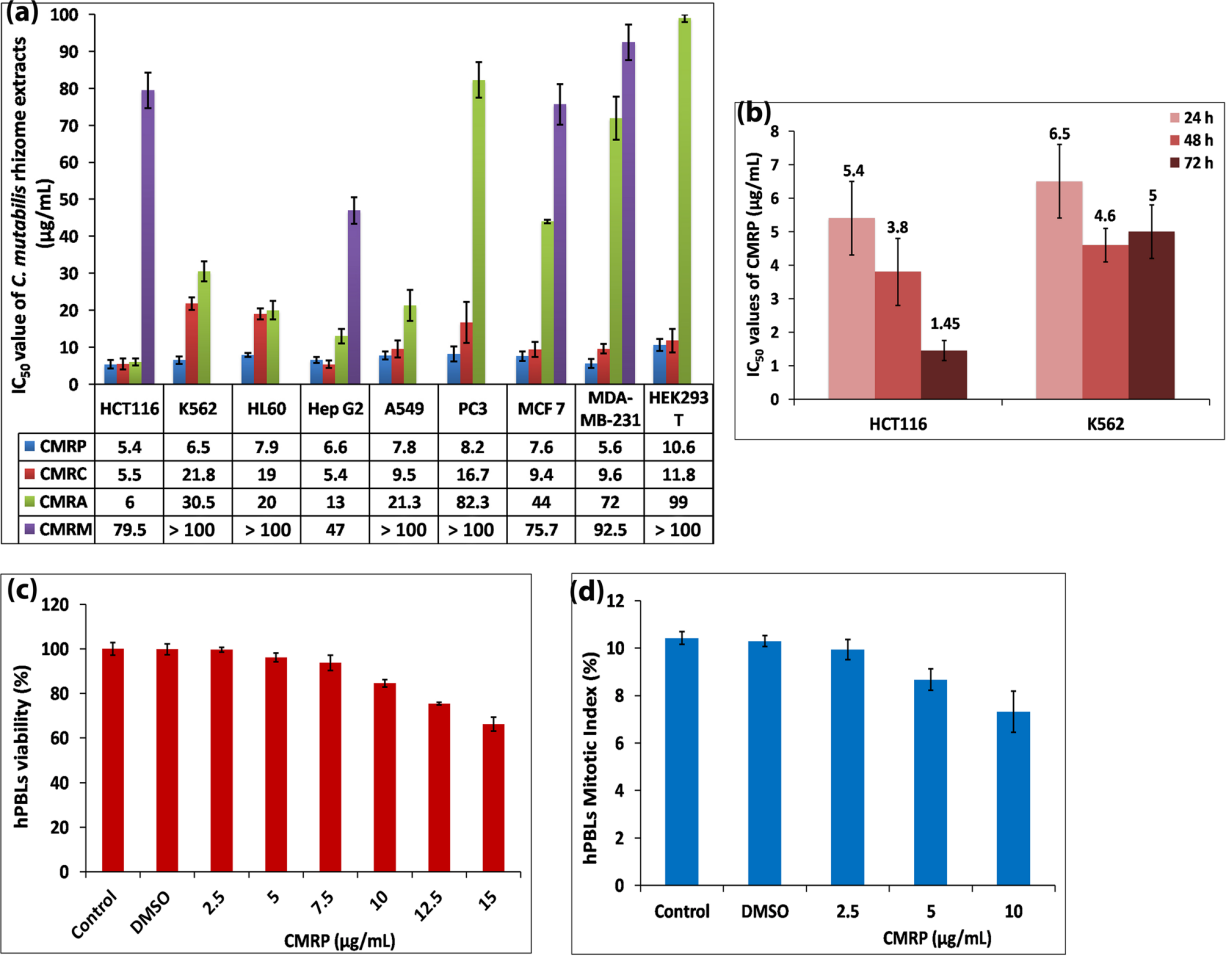
Cytotoxicity of *Curcuma mutabilis* rhizome (CMR) extracts on human cancer cell lines and normal hPBLs—(**a**) IC_50_ values of *C. mutabilis* rhizome extracts (CMRP, CMRC, CMRA and CMRM) for a 24 h treatment period against different human cancer cell lines; HCT116, K562, HL60, Hep G2, A549, PC3, MCF 7, MDA-MB-231 and embryonic kidney HEK293T cells assessed by using MTT assay. (**b**) IC_50_ values obtained for colorectal HCT116 and chronic myelogenous leukemic K562 cells following treatment with CMRP extract for 24, 48 and 72 h. (**c**) Assessment of cell viability of normal hPBLs by the MTT assay and (d) Mitotic index of cultured hPBLs. Values represent mean ± SD of three independent experiments; *P* < 0.05.

The cellular toxicities of *C. mutabilis* rhizome (CMR) extracts—petroleum ether (CMRP), chloroform (CMRC), acetone (CMRA) and methanol (CMRM) were evaluated by MTT assay using eight different human cancer cell lines such as HCT116 (colorectal carcinoma), K562 (chronic myelogenous leukemia), HL60 (acute promyelocytic leukemia), HepG2 (hepatocellular carcinoma), A549 (non-small lung alveolar carcinoma), PC3 (prostate cancer), MCF7, MDA-MB-231 (breast cancer) and embryonic kidney cells, HEK293T. All extracts showed dose-dependent reduction in cell survival percentage (Supplementary Fig. S1), with CMRP exhibiting maximum cytotoxicity against all of the 9 cell lines, with the lowest IC_50_ values ranging from 5.4–10.6 µg/mL for a 24 h treatment period followed by CMRC (5.4–21 µg/mL), CMRA (6–99 µg/mL) and CMRM (47– > 100 µg/mL) extracts (Fig. 1a,b). Trypan blue dye exclusion assay also corroborated well with the results of MTT assay (Supplementary Table S1). CMRP was found to display superior cytotoxicity/antiproliferative activity amongst all *C. mutabilis* rhizome extracts tested. Hence, all subsequent investigations to unravel mechanistics of its cellular and molecular action were carried out on two cell lines representing adherent and suspension cell types, namely, HCT116 (IC_50_–5.5 µg/mL) and K562 (IC_50_–6.5 µg/mL) which showed the highest sensitivity towards CMRP.

### CMRP is not cytotoxic to normal human peripheral blood lymphocytes (hPBLs) and erythrocytes

The results of hemolytic (< 1%) and MTT assays of CMRP-treated (2.5–15 µg/mL) normal hPBLs revealed that the extract was innocuous with no significant cytotoxicity apparent within the range of IC_50_ values (5–7.5 µg/mL) for majority of the cancer cell lines tested (Fig. 1c). Analysis of mitotic index (MI) of the CMRP-treated lymphocyte cultures along with untreated controls were found to be in agreement with MTT-based determination of percentage cell survival (Fig. 1d).

### CMRP inhibits colony-formation ability of cancer cells

**Figure 2:**
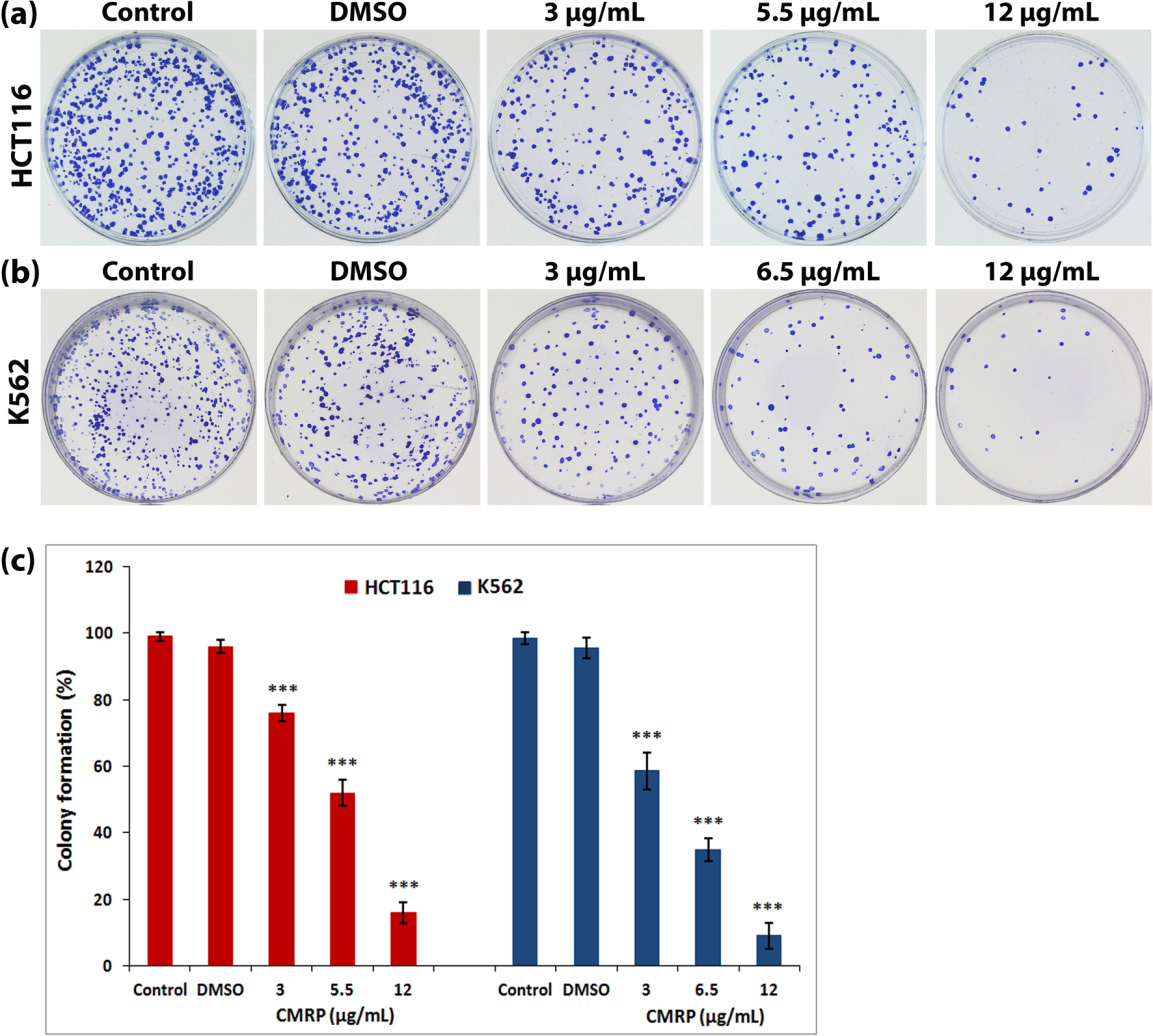
Clonogenic survival assay following treatment with CMRP. Clonogenic capacity of (**a**) HCT116 and (**b**) K562 cells treated with CMRP, following staining with crystal violet and (**c**) histogram showing significant percentage reduction in number of colonies compared to untreated and vehicle (DMSO)-treated controls. Values represent mean ± SD of three independent experiments; ***P < 0.001.

Exposure of HCT116 and K562 cells to CMRP resulted in inhibition of their colony-forming capability, in a concentration dependent manner, which was indicative of the extract’s ability to affect cell survivability and proliferation. In both cell types, CMRP treatment at a concentration of 12 µg/mL, higher than the respective IC_50_ value, resulted in severe reduction of about 80–88% in the number of colonies compared to controls (Fig. 2).

### CMRP induces apoptosis-related cytomorphological changes in cancer cells

**Figure 3:**
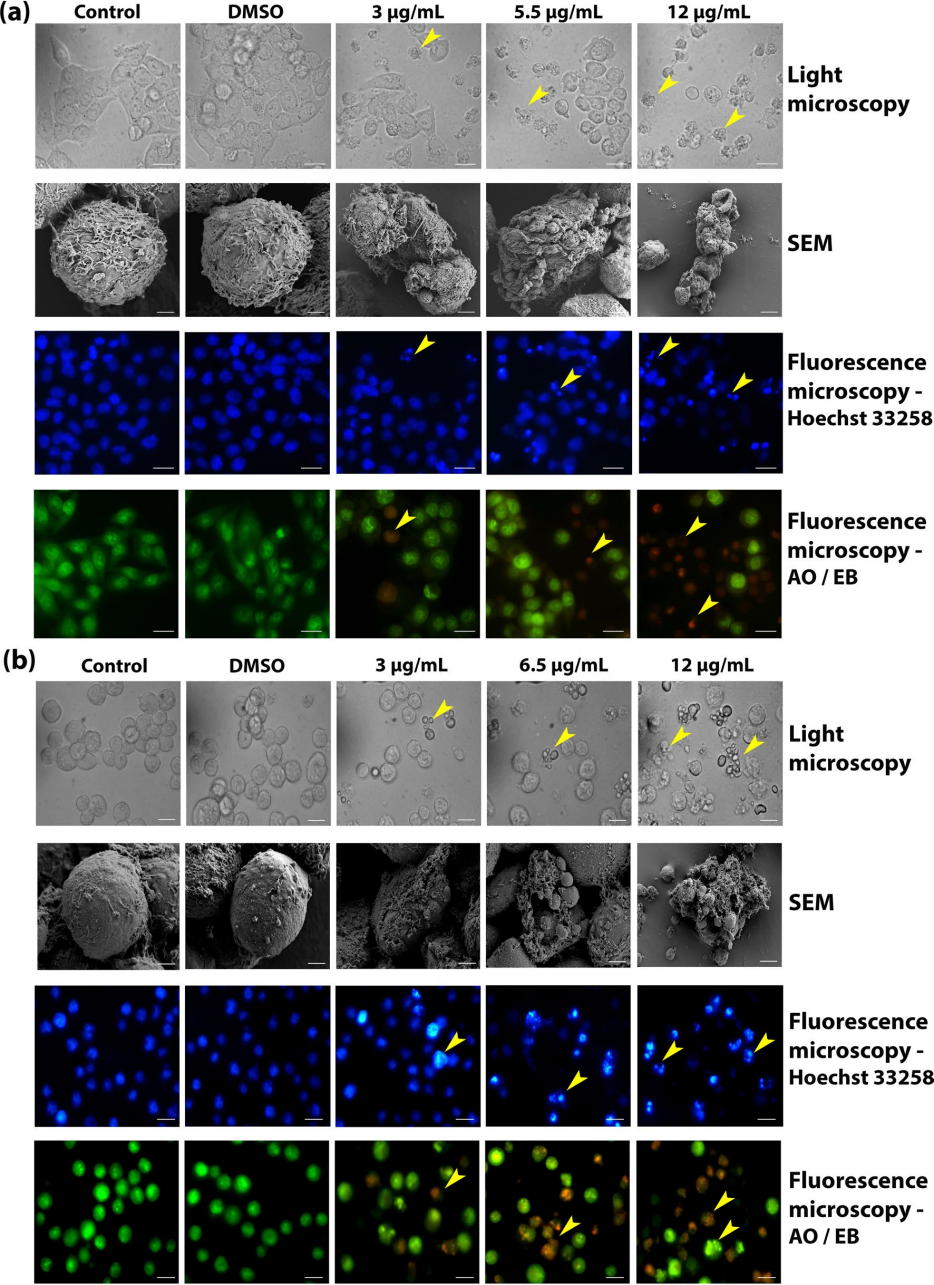
CMRP-induced cytomorphological alterations associated with apoptosis induction detected using light, scanning electron and fluorescence microscopy. (**a**) HCT116 cells and (**b**) K562 cells treated with CMRP for 24 h, following observations under light, SEM and fluorescence microscopy (Hoechst 33258 and AO/EB dual stained cells), for apoptosis- related cytomorphological changes (arrow heads indicate cells undergoing apoptosis). Scale bar: 15 µm for K562 and 20 µm for HCT116 cells for all images except for SEM (scale bar: 2 μm).

Both HCT116 and K562 cells displayed cytomorphological alternations associated with apoptosis induction, in a dose dependent manner, as observed by light and scanning electron microscopy (Fig. 3). Cell shrinkage, extensive membrane-blebbing and cellular fragmentation into apoptotic bodies were observed in K562 cells. Confluent aggregates of untreated polygonal HCT116 cells were observed to round off, shrink with membrane blebs and become detached from the culture dish surface, following a 24 h treatment with CMRP.

Fluorescence microscopy of CMRP treated cells stained with DNA binding dye, Hoechst 33258, showed a concentration-dependent increase in the number of cells which displayed bright blue, condensed and fragmented nuclei compared to the uniformly blue stained nuclei observed in untreated and vehicle (DMSO) treated controls (Fig. 3). CMRP treated cells, dual-stained with acridine orange (AO) and ethidium bromide (EB), were also found to display the characteristic staining pattern confirming induction of apoptosis in a concentration dependent manner (Fig. 3). AO is permeable across intact cell membranes and stains nuclei green, whilst EB can enter only in cells with disrupted membrane integrity to interact with DNA and emit yellow to orange fluorescence, depending upon the stage of apoptosis^16^.

### CMRP induces phosphatidylserine (PS) externalization, increase in intracellular ROS, Ca^2+^ and loss of mitochondrial membrane potential

**Figure 4:**
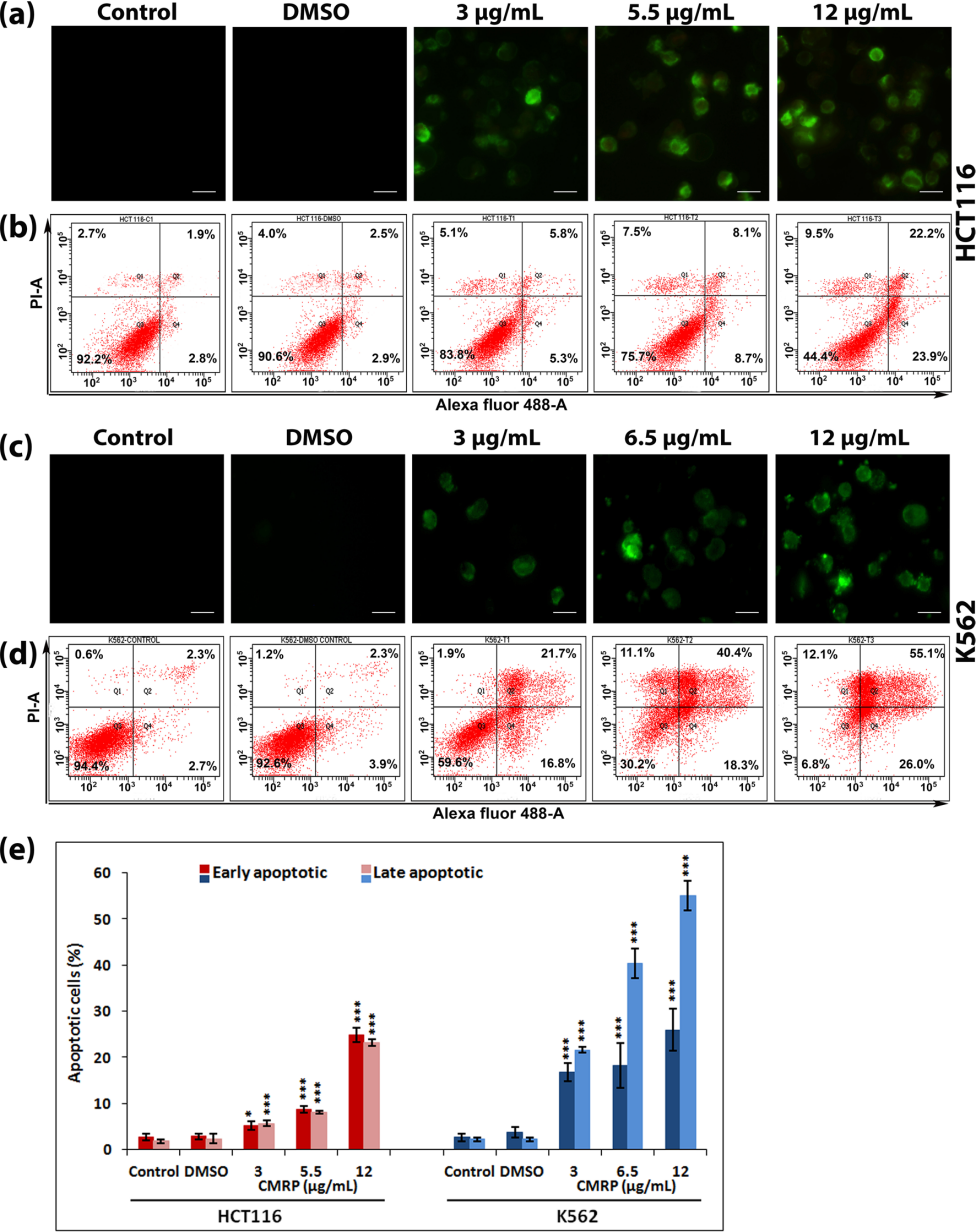
Evaluation of CMRP-induced phosphatidylserine externalization in cancer cells using Alexa Fluor 488-annexin V and PI staining. (**a**) and (**c**) fluorescence microscopy of annexin V stained HCT116 and K562 cells respectively (**b**) and (**d**) flow cytometric evaluation of annexin V and PI stained HCT116 and K562 cells: in each panel the lower left quadrant shows viable cells (negative for both annexin V and PI), lower right quadrant with only annexin V^+^ cells (early apoptotic), upper right quadrant shows annexin V^+^ and PI^+^ cells (late apoptosis) and upper left quadrant shows only PI^+^ cells (necrotic). (**e**) Histogram showing percentages of early and late apoptotic cells. *P < 0.05, ***P < 0.001.

Appearance of phosphatidylserine (PS) at cell surface, which is normally restricted to inner membrane layer, is considered as a hallmark of apoptosis. Calcium-dependent binding of fluorochrome-tagged annexin V to externalized PS enables its detection by fluorescence microscopy and quantitation using flow cytometry. Annexin V-Propidium iodide (PI) co-staining also helps to distinguish late apoptotic/necrotic cells from early apoptotic cells, as PI can enter cells and interact with DNA only when membrane integrity is lost^17^. Assessment of PS externalization in cells treated with CMRP for 24 h by flow cytometry showed an increase in the percentage of apoptotic cells in a dose dependent manner. At the highest test concentration of 12 µg/mL, late apoptotic (annexin V^+^/PI^+^) cell population increased to ~ 22 and 55% in HCT116 and K562 cells respectively (Fig. 4).

**Figure 5:**
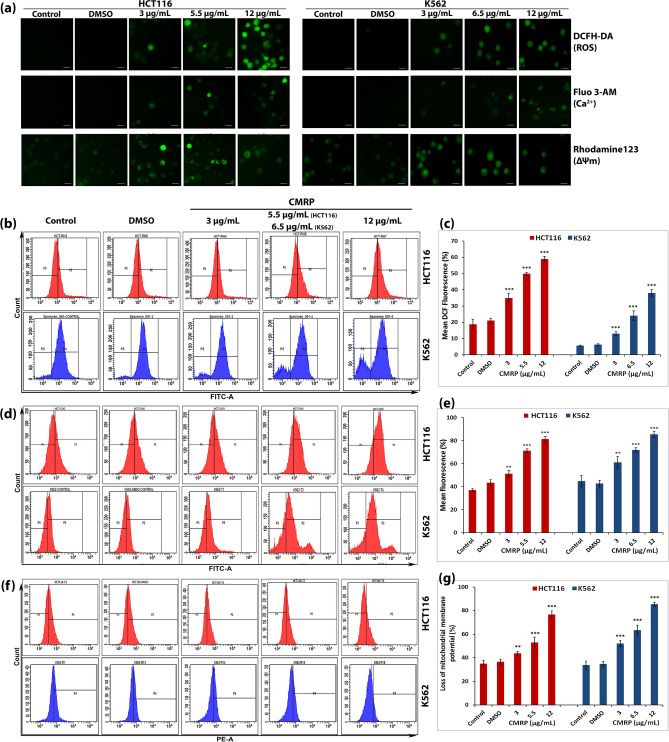
CMRP induces increase in intracellular ROS, Ca^2+^ and loss of mitochondrial membrane potential. (**a**) Fluorescence microscopy of DCFH-DA, Fluo-3 AM and Rhodamine 123 stained HCT116 and K562 cells. (**b**), (**d**) and (**f**) flow cytometric analysis for evaluation of increase in intracellular ROS, Ca^2+^ and loss of mitochondrial membrane potential respectively, while (**c**), (**e**) and (**g**) are representative histograms for mean fluorescence data obtained from flow cytometry. **P < 0.01, ***P < 0.001.

Intracellular reactive oxygen species (ROS) was assessed using DCFH-DA, a cell permeable non-fluorescent probe. Upon entry into the cell, DCFH-DA is converted into membrane impermeable H_2_DCF by cellular esterases and later oxidized by ROS into highly fluorescent DCF. Therefore, increase in its fluorescence is directly proportional to the amount of intracellular ROS^18^. In the present study, both HCT116 and K562 cell types revealed a dose dependent increase in intracellular ROS by fluorescence microscopic (Fig. 5a) and flow cytometric (Fig. 5b,c) evaluation following CMRP treatment for 24 h. Flow cytometric analysis revealed around six-fold increase in mean DCF fluorescence of K562 and nearly three-fold hike in that of HCT116 cells compared with untreated and DMSO treated controls. Likewise, assessment of intracellular Ca^2+^ levels was carried out by using cell permeant Ca^2+^ binding Fluo-3 AM probe^19^. Flow cytometric data obtained from HCT116 and K562 cells, following 24 h exposure to CMRP, clearly showed a dose dependent increase in intracellular Ca^2+^ levels (Fig. 5a,d,e).

Change in the mitochondrial membrane potential, associated with intrinsic cell death pathway, was studied using mitochondria-specific, voltage-dependent fluorescent probe Rhodamine-123^20^. CMRP-induced loss of mitochondrial membrane potential was found to occur in a dose dependent manner as evidenced by both fluorescence microscopy (Fig. 5a) and flow cytometric data (Fig. 5f,g).

### CMRP induces changes in cell cycle distribution and increase in sub-G1 population without causing cell cycle arrest

**Figure 6:**
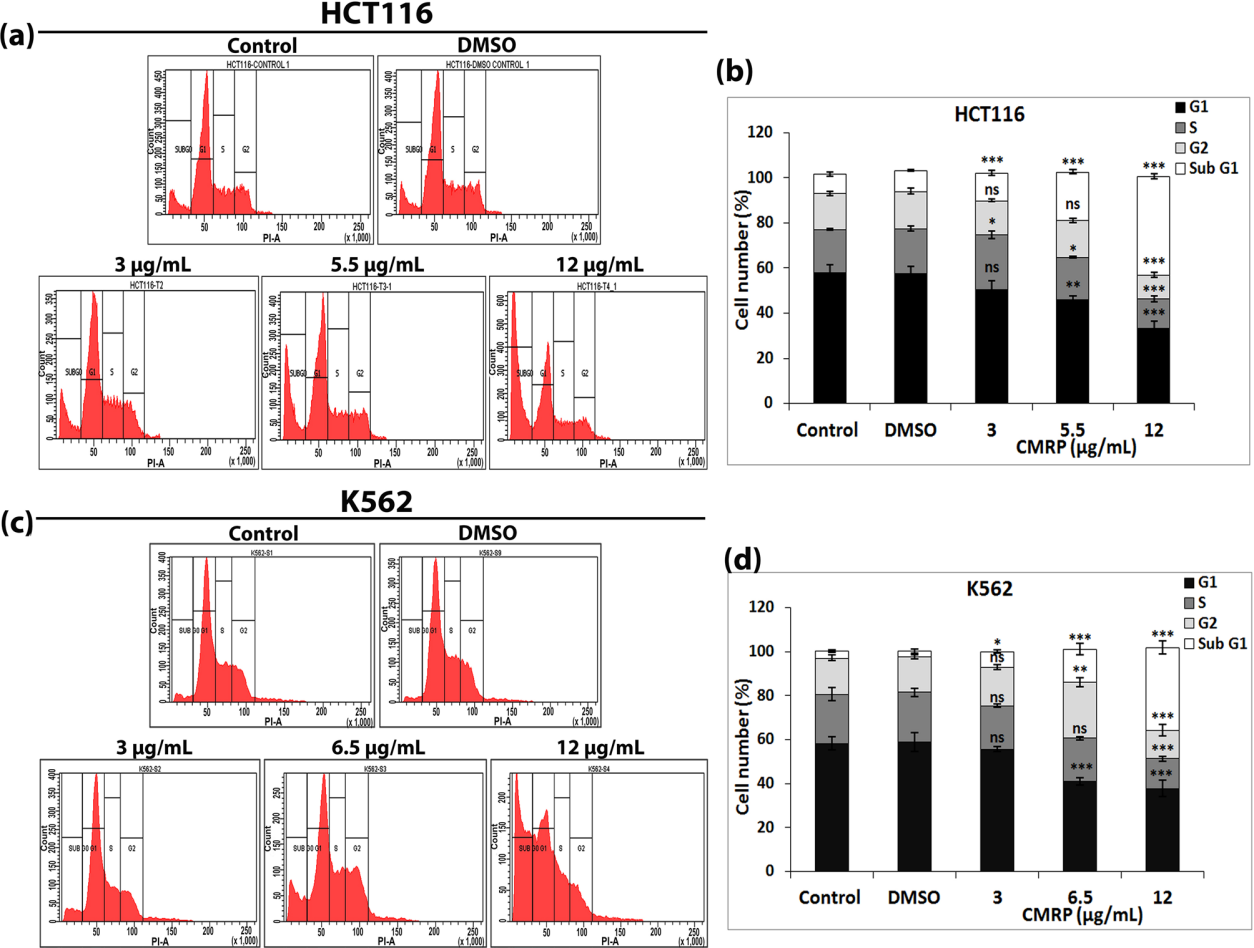
Effect of CMRP on cell cycle phase-specific distribution of cancer cells. (**a**) and (**c**) Flow cytometric analysis of cell cycle phase-specific distribution, following 24 h treatment with CMRP against HCT116 and K562 cells respectively. (**b**) and (**d**) Bar diagrams showing percentage of cells in different phases (G1, S, G2 and sub G1) of cell cycle of HCT116 and K562. Ns—not significant, *P < 0.05, **P < 0.01, ***P < 0.001.

Flow cytometric analysis of CMRP treated cells, stained with DNA-binding fluorescent dye, propidium iodide (PI), revealed changes in cell distribution in three major phases of cell cycle, G1, S and G2/M. These changes were dose-dependent and commensurate with a concomitant increase in sub-G1 apoptotic cell population harbouring fractional DNA content (Fig. 6). In other words, cell killing ability of the extract was not differential with respect to specific cell cycle phases. Any evidence of CMRP-induced cell cycle arrest at G1 or G2/M phase was also found lacking.

### CMRP induces DNA damage in cancer cells

**Figure 7:**
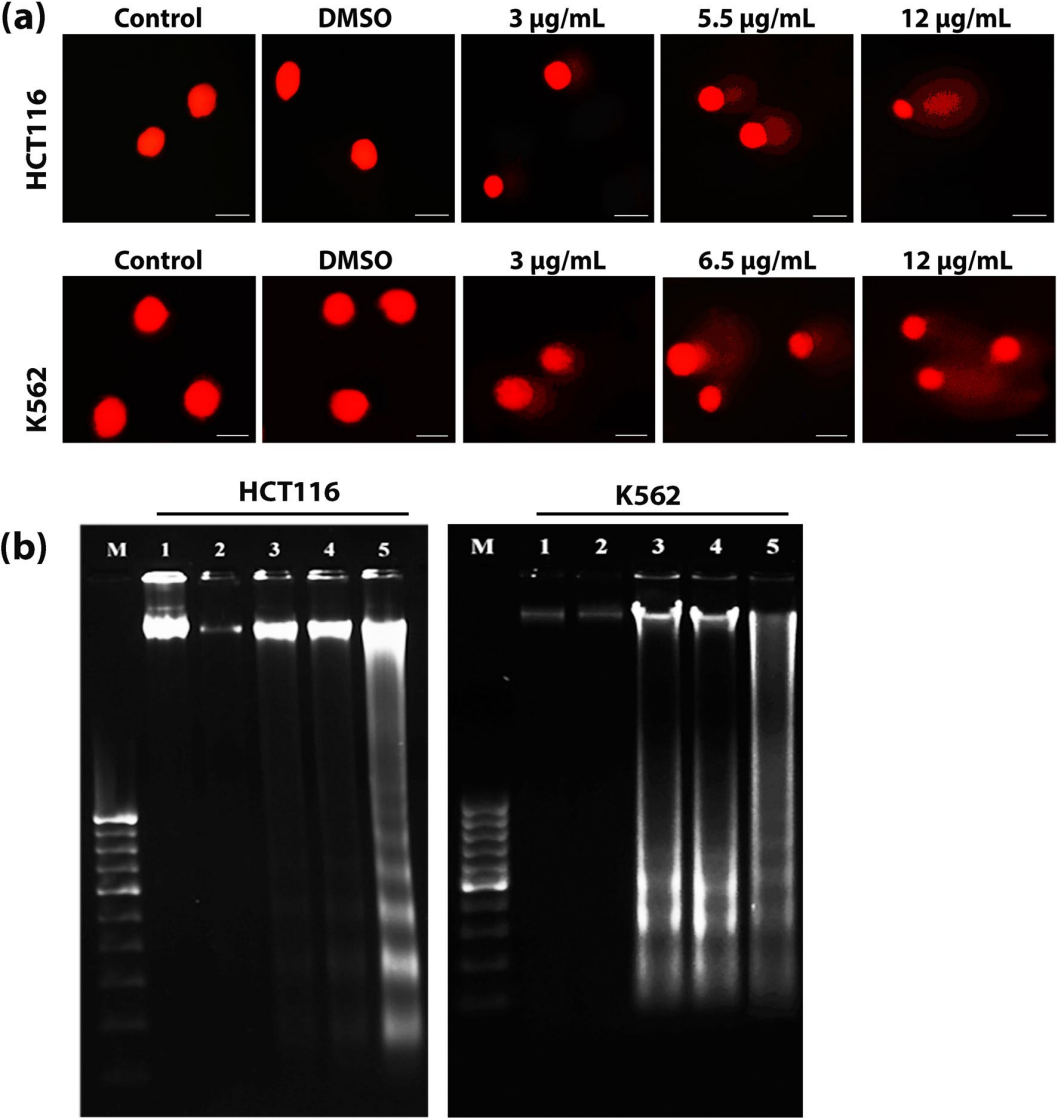
Assessment of CMRP-induced DNA damage in HCT116 and K562 cancer cells. (**a**) Genotoxicity evaluation by alkaline comet assay. (**b**) DNA fragmentation assay, Lane M: 100 bp DNA ladder, Lane 1: DNA from control cells, Lane 2: DNA from DMSO-treated cells, Lane 3: DNA from CMRP (3 µg/mL) treated cells, Lane 4: DNA from CMRP (5.5 µg/mL for HCT116 and 6.5 µg/mL for K562) treated cells, Lane 5: DNA from CMRP (12 µg/mL) treated cells. Full length agarose gels with fragmented DNA are included in Supplementary Figure S9.

Occurrence of DNA strand breaks within cancer cells was detected by comet assay (single cell electrophoresis), a sensitive, reliable and rapid method for genotoxic assessment^21^. CMRP treated HCT116 and K562 cells displayed increased comet tail lengths, in a concentration dependent manner, indicative of the occurrence of DNA damage (Fig. 7a). Likewise, DNA fragmentation/laddering, a biochemical hallmark of apoptosis, was also detected in DNA isolated from the CMRP-treated cells, analysed on 1.8% agarose gels (Fig. 7b).

### CMRP induces changes in apoptosis-related gene expression

**Figure 8:**
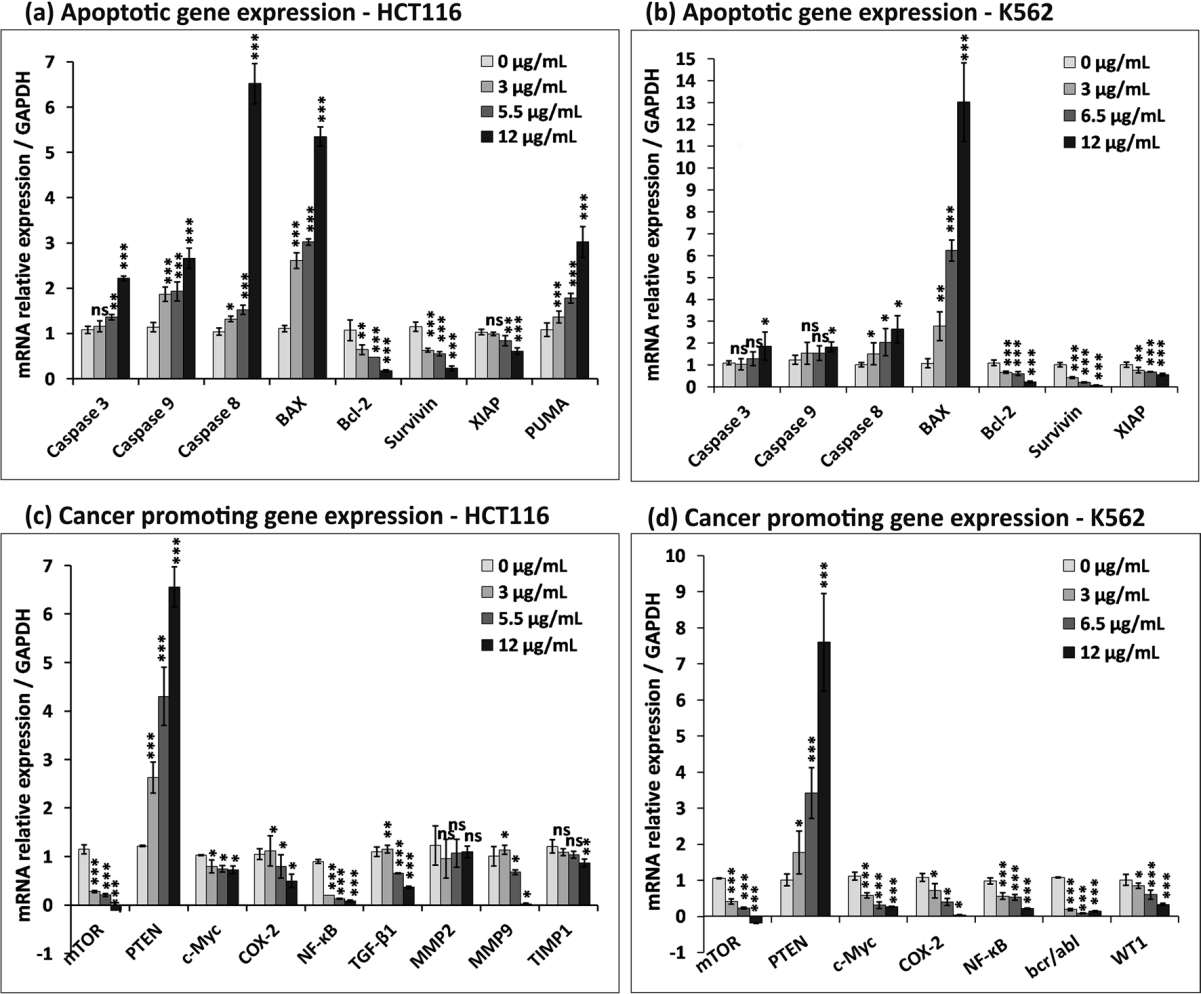
Quantitative transcriptional profiling of CMRP treated K562 and HCT116 cells using RT-qPCR. (**a**) and (**b**) Bar diagram representing fold-changes in the relative expression of mRNA from apoptotic genes and (**c**) and (**d**) those from cancer promoting genes, normalized to that of GAPDH gene. Data represents mean ± SD of three independent experiments; ns—not significant, *P < 0.05, **P < 0.01, ***P < 0.001.

Molecular analysis of CMRP-induced changes in cancer cells was carried out by RT-qPCR and western blot analysis. The levels of apoptosis-related gene transcripts, caspases -3, -8 and -9, pro-apoptotic BAX and PUMA, anti-apoptotic Bcl-2 and apoptosis inhibitors, survivin and XIAP, were studied. Figure 8 a,b shows the expression profiling data from HCT116 and K562 cells, normalized to the housekeeping (reference) gene, GAPDH. No significant fold changes in transcription were observed for caspase -3 and -9 in both cell types, except caspase-8, which showed a six fold increment in HCT116 treated with CMRP at 12 µg/mL, beyond the IC_50_ value. Likewise, upregulation of pro-apoptotic BAX gene expression was evident in K562 cells by ~ 6 and 13 folds respectively in cells treated at the IC_50_ value (6.5 µg/mL) and beyond (12 µg/mL). HCT116 cells, exposed to 5.5 µg/mL and 12 µg/mL showed a 3 and 5-folds increase respectively in BAX expression relative to controls. PUMA expression was also found to be upregulated by 3 folds in HCT116 cells at 12 µg/mL. As expected, the anti-apoptotic Bcl-2 gene expression was found to be downregulated in both cell types by about 0.18 and 0.22 folds at 12 µg/mL, in a concentration dependent manner. Given the fact that survivin and XIAP are inhibitors of apoptosis (IAP), their expression was indicative of promotion of cancer cell survival^22^. CMRP treatment was found to cause reduction in survivin and XIAP expression in a dose dependent manner. Notably, the effect of extract on survivin downregulation was greater than that on XIAP, especially in K562 cells with only 0.07 folds expression at 12 µg/mL. Overall, CMRP treatment resulted in upregulation of pro-apoptotic BAX and downregulation of anti-apoptotic Bcl-2 and IAPs (survivin and XIAP) gene expressions. The extent of caspase gene expression in both cell types was also found to be upregulated, more so for caspase 8 in HCT116 cells, by CMRP treatment. In other words, CMRP treatment apparently activated expression of genes involved in both intrinsic and extrinsic apoptotic pathways.

**Figure 9:**
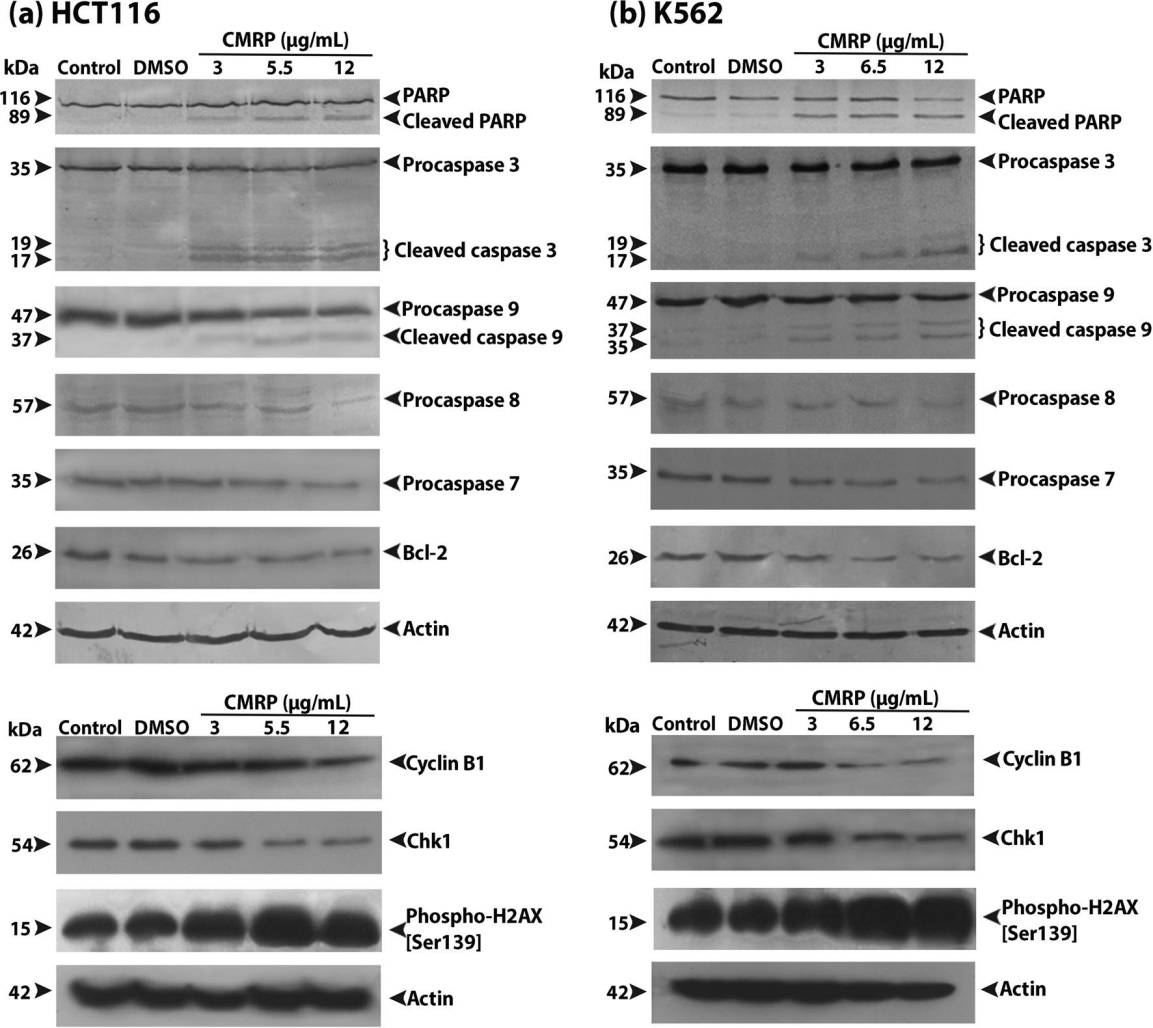
Effect of CMRP on expression of apoptosis and cell cycle related proteins in cancer cells analysed by western blotting. (**a**) and (**b**) Proteins (~ 50 μg) extracted from control and treated HCT116 and K562 cells was resolved on SDS-PAGE followed by western blot analysis using antibodies against PARP, caspase -3, -7, -8, -9, Bcl-2, cyclin B1, Chk 1, and γ-H2AX. β-actin was taken as the loading control. Full length western blots were displayed in Supplementary Figure SS10.

Western blot analysis of CMRP treated HCT116 and K562 cells revealed cleavage of PARP, procaspases -3 and -9, in a dose dependent manner, indicative of apoptosis induction (Fig. 9). A marginal reduction in the expression of procaspases -7 and -8 relative to controls and downregulation of anti-apoptotic Bcl-2 was also apparent. On the whole, the results of immunoblotting clearly confirmed CMRP-induced activation of both intrinsic and extrinsic pathways of apoptosis. CMRP-induced increase in the levels of the phosphorylated form of histone variant, γ-H2AX, provided compelling and corroborative evidence of DNA damage, whilst reduction in cyclin B1 and Chk1 expression was indicative of the extract’s ability to influence the cell cycle of cancer cells.

### CMRP influences expression of genes essential for cell survival

RT-qPCR data obtained from HCT116 and K562cells also revealed CMRP-induced variations in the transcript levels of some of the key genes involved in cell survival, cancer progression and migration (Fig. 8c,d). Expression of mTOR and PTEN involved in PI3K/Akt/mTOR pathway was found to be affected by CMRP; at a high concentration of 12 µg/mL, mTOR expression was drastically reduced, with an increase in PTEN expression. In K562, the levels of expression of the proto-oncogene, c-Myc, were considerably reduced by CMRP treatment, without any significant change induced in HCT116 cells. Likewise, COX-2 and NF-κB subunit RelA (p65) gene expressions were found to be highly downregulated by treatment in both cell types. A 24 h CMRP treatment was also found to cause a dose dependent downregulation of leukemic *bcr-abl* and *WT1* gene expression in K562 cells. A drastic reduction in the expression of TGF-β1and MMP-9 proteins known to be involved in epithelial to mesenchymal transition (EMT) and tumour cell migration was also observed in CMRP-treated HCT116 cells.

### CMRP inhibits cell migration and invasion of HCT116

CMRP-treated HCT116 cells were observed to move slower than the untreated / DMSO-treated controls in a dose-dependent manner (Supplementary Fig. S2), as evidenced by the results of in vitro scratch assay. Also, a dose dependent inhibition of invasive ability in HCT116 cells was clearly visualized by transwell (coated with Geltrex matrix) invasion assay.

### Isolation, characterization and identification of active principle(s) of CMRP

The CMRP extract was sub-fractionated on silica gel columns as described in the Methods section. From a total of 55 sub-fractions (SF) (Supplementary Table S2), 19 fractions [SF16-24: IC_50_ ≤ 2.5 µg/mL; SF25-28: IC_50_ 3–17.5 µg/mL and SF32: IC_50_ 16–28 µg/mL] which displayed IC_50_ values < 30 µg/mL were preselected for TLC analysis. Guided by the TLC banding patterns, the three sub-fractions designated SF20, -27 and -32 (Supplementary Fig. S3) were analysed further for identification of the compounds. TLC (toluene:ethyl acetate (7:3, *v/v*): R_f_ = 0.74), UV–visible spectroscopy and HPLC analysis together revealed that SF20 contained a single component, which was identified later as a labdane diterpenoid, namely, (*E*)-14,15-epoxylabda-8(17),12-dien-16-al (IUPAC) and denoted as ‘*Curcuma mutabilis* epoxide’ (Cm epoxide) by us.

### Cm epoxide structure

Analysis of the data obtained using HRESIMS, FTIR and NMR spectra (Supplementary Fig. S4) described below revealed the structure of Cm epoxide. Mass spectroscopic data indicated that Cm epoxide has a molecular formula of C_30_H_20_O_2_, as supported by HRESIMS [M + Na]^+^ at *m/z* 325.215 or [M]^+^ at *m/z *302.226. FTIR spectra of Cm epoxide showed absorption bands at (cm^−1^) 1166 and 1248.6 (C–O–C stretching within the ether and epoxide groups respectively), 1692.2 (C = O stretching associated with –CHO group), 1649.8 (alkenyl C = C stretch in conjugation with –CHO group), 2706.6 (aldehyde), 2924.5 and 2839.6 (C–H stretching of alkanes in cyclohexyl ring), 1384.6 (gem-dimethyl group), 896.7 (vinylidene C–H group). ^1^H NMR spectra (600 MHz, CDCl_3_): δ (ppm) 0.759 (3H, s), 0.827 (3H, s), 0.892 (3H, s), 2.026 (1H,ddd, *J* ) 13.05, 13.05, 5.14 Hz), 2.417 (1H, ddd, *J* ) 12.90, 4.34, 2.38 Hz), 2.642 (1H, ddd, *J* ) 16.84, 11.33, 7.54 Hz), 2.744 (1H, ddd, *J* ) 16.83, 6.10, 3.05 Hz), 2.872 (1H, dd, *J* ) 5.55, 2.79 Hz), 3.035 (1H, dd, *J* ) 5.55, 4.39 Hz), 3.657 (1H, dd *J* ) 3.58 Hz), 4.468 (1H, s), 4.865 (1H, s), 6.624 (1H, dd, *J* ) 7.53, 6.17 Hz), 9.329 (1H, s); 13C NMR (150.9 MHz, CDCl_3_): δ 193.45, 161.15, 148.19, 137.55, 107.86, 56.89, 55.46, 47.42, 47.09, 42.04, 39.71, 39.18, 37.93, 33.60, 33.59, 24.22, 24.18, 21.74, 19.31, 14.45 (Details of NMR data are given in Table 1).

**Table 1:** Comparison of ^1^H and ^13^C NMR data of SF20 (Cm epoxide) from *Curcuma mutabilis* with Alpinia epoxide reported from *Alpinia chinensis* in CDCl_3_.

Atom	^13^C *δ* (ppm) (literature)	^13^C *δ* (ppm) (SF20)	Δ*δ* (ppm)	^1^H *δ* (ppm); integ. ≠ 1; mult.;*J* (Hz) (literature)	^1^H *δ* (ppm); integ. ≠ 1; mult.;^a^*J* (Hz)^b^ (SF20)	Δ*δ* (ppm)
1*β*	39.1	39.18	+ 0.08	1.75; –; –	1.750;ovlm	0.00
1*α*				1.10; –; –	1.109;td;*J*_H1*β*_, − 12.86;*J*_H2*β*_, 12.86;*J*_H2*α*_, 3.90	+ 0.009
2*β*	19.3	19.31	+ 0.01	1.60; –; –	1.59;qt_AB_t;*J*_H2*α*_, − 13.61;*J*_H3*α*_ = *J*_H1*α*_, 13.61;*J*_H3*β*_ = *J*_H1*β*_, 3.41	− 0.010
2*α*				1.53; –; –	1.52;m	− 0.010
3*β*	42.0	42.04	+ 0.04	1.42; –; –	1.419;d_AB_td;*J*_H3*α*_, − 13.24;*J*_H2*β*_ = *J*_H2*α*_, 3.27;*J*_H1*β*_, 1.57	− 0.001
3*α*				1.20; –; –	1.201;td;*J*_H3*β*_, − 13.40;*J*_H2*β*_, 13.40;*J*_H2*α*_, 4.14	+ 0.001
4	33.5	33.59	+ 0.09	–	–	–
5	55.3	55.46	+ 0.16	1.15; –; –	1.148;dd;*J*_H6*β*_, 12.65;*J*_H6*α*_, 2.81	− 0.002
6*β*	24.1	24.18	+ 0.08	1.35; –; –	1.360;qtd;*J*_H6*α*_, − 12.97;*J*_H5_ = *J*_H7*α*_, 12.97;*J*_H7*β*_, 4.32	+ 0.010
6*α*				1.75; –; –	1.754;ovlm	+ 0.004
*7β*	37.9	37.93	+ 0.03	2.40;ddd; 12.8; 4.3; 2.3	2.417;ddd;*J*_H7*α*_, − 12.90;*J*_H6*β*_,4.34;*J*_H6*α*_,2.38	+ 0.017
7*α*				2.02;ddd; 12.9; 12.8; 5.0	2.026;td(tdm);*J*_H7*β*_, − 13.05;*J*_H6*α*_,13.05;*J*_H6*β*_,5.14	+ 0.006
8	148.0	148.19	+ 0.19	–	–	–
9	56.8	56.89	+ 0.09	1.90; –; –	1.886;dbrd (dm);*J*_H11*proR*_, 11.39	− 0.014
10	39.6	39.71	+ 0.11	–	–	–
11_*proS*_	24.1	24.22	+ 0.12	2.75;ddd; 16.8; 6.0; 3.0	2.744;d_AB_dd;*J*_H11*proR*_, − 16.83;*J*_H12_, 6.10;*J*_H9_,3.05	− 0.006
11_*proR*_				2.65;ddd; 16.8; 11.2; 7.5	2.642;d_AB_dd;*J*_H11*proS*_, − 16.84;*J*_H9_,11.33;*J*_H12_,7.54	− 0.008
12	160.6	161.15	+ 0.55	6.61;dd; 7.5; 6.0	6.624;dd(ddd);*J*_H11*proR*_, 7.53;*J*_H11*proS*_, 6.17;*J*_H14_, 0.59	+ 0.014
13	137.5	137.55	+ 0.05	–	–	–
14	47.3	47.42	+ 0.12	3.63;dd; 4.5; 2.7	3.658;t(~ tm);*J*_H15*proS*_,_H15*proR*_ (aver.), 3.58	+ 0.028
15_*proS*_	46.2	47.09	+ 0.89	2.99; dd; 5.5; 4.5	3.035; dd; *J*_H15*proR*_, 5.55; *J*_H14_, 4.39	+ 0.032
15_*proR*_				2.84; dd; 5.5; 2.7	2.872; dd; *J*_H15*proS*_, 5.55; *J*_H14_, 2.79	+ 0.045
16	193.2	193.45	+ 0.25	9.31; s	9.329;s (d);*J*_H14_, 0.37	+ 0.019
17_*Z*_	107.8	107.86	+ 0.06	4.48; s	4.468;qt; *J*_H17*E*_ = *J*_H7*α*_ = *J*_H9_, 1.44	− 0.012
17_*E*_				4.86; s	4.865;qt (m)	+ 0.005
18	33.6	33.60	0.00	0.89; 3H; s	0.892;3H; s	+ 0.002
19	21.7	21.74	+ 0.04	0.83; 3H; s	0.827;3H; s	− 0.003
20	14.4	14.45	+ 0.05	0.76; 3H; s	0.759;3H; s (d);*J*_H1*α*_, 0.65	− 0.001

^a^Multiplicities in parentheses after application of an exponential (− 0.7 Hz)–Gaussian (+ 0.3 Hz) window function.

^b^Large geminal couplings assumed to be negative.

**Figure 10:**
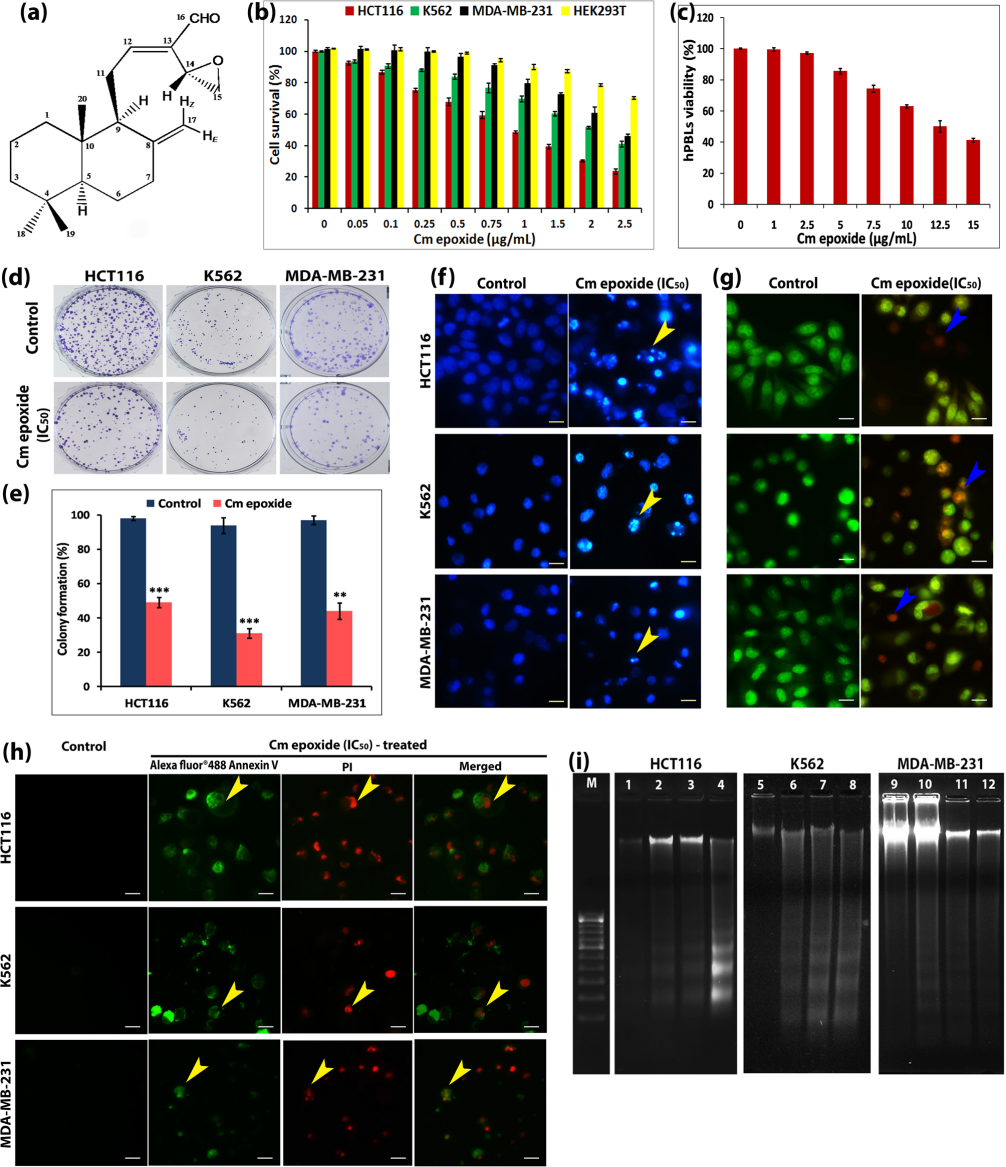
Evaluation of cytotoxicity and apoptogenic potential of Cm epoxide. (**a**) Chemical structure of (*E*)-14,15-epoxylabda-8(17),12-dien-16-al (Cm epoxide). (**b**) and (**c**) Evaluation of cell survival / viability percentage of Cm epoxide treated (24 h) cancer cells (HCT116, K562, MDA-MB-231 and HEK293T) and hPBLs respectively using MTT assay; values represent mean ± SD of three independent experiments; *P* < 0.05. (**d**) Clonogenic capacity of HCT116, K562 and MDA-MB-231 cancer cells treated with IC_50_ concentrations of Cm epoxide and stained with crystal violet. (**e**) Histogram showing significant percentage reduction in number of colonies compared to untreated controls. **P < 0.01,***P < 0.001. (**f**) and (**g**) Fluorescence microscopy of Hoechst 33258 and AO / EB dual stained cells for apoptosis-related cytomorphological changes. (**h**) Alexa Fluor 488 annexin V-PI co-stained cells for phosphatidylserine externalization analysis, arrow heads indicate apoptotic cells (Scale bar : 10 µm). (**i**) Cm epoxide-induced DNA fragmentation in HCT116, K562 and MDA-MB-231 cells—Lane M :100 bp DNA ladder; Lane 1, 5 and 9: DNA from untreated control cells (in all cell lines); Lane 2, 3 and 4: DNA from HCT116 cells treated with 0.5, 0.9 and 2.5 µg/mL of Cm epoxide respectively; Lane 6,7 and 8: DNA from K562 cells treated with 1.0, 2.0 and 5.0 µg/mL of Cm epoxide respectively; Lane 10, 11 and 12: DNA from MDA-MB-231 cells treated with 1.0, 2.5 and 5.0 µg/mL of Cm epoxide respectively. Full length agarose gels with DNA fragmentation were given in Supplementary Figure S11.

^13^C/DEPT NMR confirmed the presence of 20 carbon atoms with 30 protons directly attached to it including an exocylic double bond (δ_C_ 107.8 CH_2_,148.2 C) in addition to the unsaturated aldehyde group (δ_C_ 193.5 CH, 137.5 C, 161.2 CH). ^1^H NMR indicating three protons of a terminal epoxide (δ_H_ 3.66, dd, *J* = 3.58 Hz, H-14; 3.03, dd, *J* = 5.5, 4.4 Hz, H-15a; 2.87, dd, *J* = 5.5, 2.8 Hz, H-15b). The standard application and interpretation of 2D—COSY, HSQC and HMBC NMR spectra (Supplementary Fig. S4) of SF20 showed its chemical formula of C_20_H_30_O_2_ (Δmass = 2.4 ppm from the calculated value) and its structure is depicted in Fig. 10a. The gross structure was readily determined with all stereochemical relationships in a methodical manner covering the relative configurations of the stereocenters, the *α, β* orientations of the ring protons, the *proR, proS* relationships of the geminal proton pairs, the *E*, *Z* orientations of the geminal proton pair, and the *E* configuration of the C_12_ = C_13_ double bond by the application of NOE (Nuclear Overhauser Effect) and scalar coupling constant data. The configuration of the distal C-14 stereocenter was determined by concerted application of NOE, ^3^*J*_H,H_, and ^3^*J*_H,C_ data (by the strong or weak correlations observed in the HMBC spectrum in the latter case). Thus the compound was identified as (*E*)-14,15-epoxylabda-8(17),12-dien-16-al.

### Characterization of SF27 and -32 by GC–MS analysis

The two bioactive sub-fractions of CMRP, SF27 and -32 displayed a distinctive pattern comprising respectively of five and four major TLC bands with R_f_ values of 0.30, 0.53, 0.65, 0.73, 0.93 and 0.18, 0.20, 0.29, 0.52 (unlike the single band observed in SF20) [Supplementary Fig. S3]. Hence, these fractions were subjected to GC- MS analysis (Supplementary Table S3) to profile the volatile/semi-volatile organic constituents present therein. SF27 was found to harbour 13-apo-beta-carotenone [(3E,5E,7E)-6-methyl-8-(2,6,6-trimethyl-1-cyclohexenyl)-3,5,7-octatrien-2-one], trilaurin [dodecanoic acid, 1,2,3-propanetriyl ester], borneol and 2,4-Di-tert-butylphenol. SF32 comprised of 13-apo-beta-carotenone, bicyclo [4.1.0] heptane, 7-bicyclo[4.1.0]hept-7-ylidene, γ-sitosterol and β-amyrin.

### Cm epoxide is cytotoxic and apoptogenic to cancer cells but not to normal human lymphocytes and erythrocytes

For cytotoxicity evaluation, three cancer cells, HCT116, K562 and MDA-MB-231, along with a human embryonic kidney cell line, HEK293T, were exposed to varying concentrations (0.05–2.5 µg/mL) of Cm epoxide for 24 h (Fig. 10b). Based on MTT assay, HCT116 displayed relatively very high sensitivity towards Cm epoxide with an IC_50_ value of 0.97 µg/mL (3.2 µM) followed by that shown by K562 [IC_50_–2.05 µg/mL (6.8 µM)], MDA-MB-231 [IC_50_–2.33 µg/mL (7.7 µM)] and human embryonic kidney cells [IC_50_–4.25 µg/mL (14 µM)]. It was interesting to note that compared to the well known polyphenol from *Curcuma* spp., curcumin, which served as positive control, the IC_50_ values of Cm epoxide (Table 2) were many folds lower than that of curcumin, thereby attesting to its extremely high cytotoxic potential. The results of trypan blue dye exclusion assay also corroborated well with that of MTT assay. Interestingly, as observed with CMRP, selective killing of cancer cells without harming normal hPBLs and erythrocytes was also a property displayed by Cm epoxide. Cytotoxicity of this compound against normal hPBLs as determined by MTT assay revealed that at the IC_50_ concentrations for cancer cell lines (< 2.5 µg/mL), it was found to be rather insignificant with < 5% cell death (Fig. 10c).

**Table 2:** IC_50_ values obtained with Cm epoxide versus curcumin for 24 h treatment period using different cell lines.

Cell lines	Cm epoxide		Curcumin		Fold change (×)
	µg/mL	µM	µg/mL	µM	
HCT116	0.97 ± 0.02	3.21	7.9 ± 1.7	21.45	8×
K562	2.05 ± 0.04	6.78	14.8 ± 1.2	40.17	7×
MDA-MB-231	2.33 ± 0.14	7.71	11.5 ± 3.0	31.22	5×
HEK293T	4.25 ± 0.5	14.06	> 100	–	> 23×

Values represent mean ± SD of three experiments; *P* < 0.05.

Cm epoxide, akin to its parent extract, CMRP, displayed relatively higher potential for clonogenic inhibition (Fig. 10d,e) and apoptogenicity against cancer cells. As mentioned earlier, apoptosis-associated cytomorphological changes were assessed using light, scanning electron (Supplementary Fig. S5c,d) and fluorescence microscopy (Fig. 10f,g). Likewise, fluorescence microscopy of Alexa Fluor 488—annexin V/PI stained cells (Fig. 10h), flow cytometric analysis of cell cycle phase-specific distribution, increased sub-population of sub-G1 phase cells (Supplementary Fig. S5e,f) and DNA laddering due to internucleosomal DNA cleavage, all provided unequivocal proof of the congruency of bioactivities of both Cm epoxide as well as its parent extract, CMRP (Fig. 10i).

### CMRP and Cm epoxide are pharmacologically safe in Swiss albino mice

Swiss albino mice model was used to evaluate the pharmacological safety of CMRP and Cm epoxide in terms of acute toxicity assessment. Any elicitation of serious behavioural changes or mortality in the animal test groups was not observed. Likewise, no significant deviations were observed in hematological parameters (hemoglobin content, erythrocyte/leukocyte counts in Supplementary Table S4), levels of liver enzymes (AST, ALT and ALP) and histopathology of hematoxylin–eosin (H-E) stained liver tissue sections obtained from the control and treated groups at the tested dosages of 50 and 200 mg CMRP/Kg body weight or 25 and 100 mg Cm epoxide/kg body weight of the animal (Supplementary Fig. 6). Cm epoxide was also found capable of docking interactions with proteins known to be crucial for cell survival and death such as Epidermal Growth Factor Receptor (EGFR, PDB ID-3POZ), B-Rapidly Accelerated Fibrosarcoma Kinase (B-RAF, 3OG7) and Cyclin Dependent Kinase 2 (CDK2, 4BCP). The docking scores were comparable to that of well known inhibitors of these proteins (such as Gefitinib, Sorafenib and Flavopiridol), thereby proving the compound’s druggability against the protein targets (Supplementary Table S5 and Fig. S7).

## Discussion

The quest for novel anticancer drugs without adverse side-effects, sourced from time-tested, experiential ancient wisdom as practiced through use of traditional or ethnomedicinal plants, continue to contribute meaningfully towards cancer cure^5,23^. The ethnomedically important Zingiberaceae family members, including those from genus *Curcuma*, have been credited with a plethora of bioactivities such as anti-inflammatory, hypocholesterolemic, antidiabetic, antimicrobial, antiviral, antioxidant and anticancerous among others^8,24^. The present study reports for the first time on the potent cytototoxic/antiproliferative activity of petroleum ether extract, CMRP, prepared from the rhizome of *C. mutabilis*, an endemic Zingiberaceae species confined to Western Ghats of Kerala in India. Bioactivity-guided fractionation of the CMRP extract using silica gel column chromatography led to the isolation of three different potent cytotoxic sub-fractions, SF20, -27 and -32. Spectroscopic analysis of SF20 by HRESIMS, FTIR and NMR techniques led to the identification of a labdane diterpenoid, namely, (*E*)-14,15-epoxylabda-8(17),12-dien-16-al, denoted as Cm epoxide by us, is being reported here for the first time from *C. mutabilis*. Incidentally, this compound has been reported from another Zingiberaceae plant *Alpinia chinensis* from Hong Kong earlier as ‘Alpinia epoxide’^25^, but proof on any bioactivity including anticancer associated with the same was found missing in scientific literature. The observed NMR data of SF20 (Table 1) agrees extremely well with the published NMR data of Alpinia epoxide, thereby confirming the assigned structure. Since the relative configurations and other stereochemical aspects were confirmed in all instances by NOEs and/or scalar coupling constants, including the configuration of C-14 in the epoxide moiety, and further confirmed by the near identical match to the reported chemical shifts, the molecule can be safely considered to have a relative configuration of 5S*,9S*,10S*,14S* as previously determined^26^. Any marginal deviations between the chemical shifts observed here and those reported previously are attributable to either temperature and/or concentration effects. Indeed, the carbon spectrum was also acquired with inverse-gated decoupling to precisely match the conditions of the DEPT spectrum (same acquisition times and post-acquisition delays) and thus enable the distinction of C-4 and C-18, the multiplicity assignments of which were otherwise ambiguous. Similarly, chemical shift deviations attributable to decoupling-induced heating in the HSQC spectrum were accounted for by also acquiring the HSQC spectrum without decoupling of the carbon nuclei. Incidentally, it may be relevant to note here that, labdane diterpenoids are bicyclic diterpenes reported with a wide range of bioactivities such as antimicrobial, anti-mutagenic, anti-inflammatory, cytotoxic and cytostatic effects^27,28^. Another labdane diterpenoid, (*E*)-labda-8(17),12-diene-15,16-dial, which displays high structural similarity with Cm epoxide is widely distributed among *Alpinia* sp. It has been reported to possess antibacterial and antifungal activities in addition to being an inhibitor of α-glucosidase and NO production^29^. Interestingly, the same compound has also been reported from *C. amada* (mango ginger) in India and is known to possess antimicrobial activities^30^. Notably, this compound is structurally different from Cm epoxide due to the presence of an aldehyde (–CHO) group at C-14 position instead of an epoxide group (Supplementary Fig. S8). Further, four new labdane diterpenoids have been isolated recently from the methanol extract of *C. amada* rhizomes collected from Myanmar. These include [1] 12β-hydroxy-15-norlabda-8(17),13(14)-dien-16-oic acid, [2] (*E*)-15-ethoxy-15-methoxylabda-8(17)12-dien-16-al, [3] (*E*)-15α-ethoxy-14α-hydroxylabda-8(17),12-dien-16-olide, and [4] 15-ethoxy-12β-hydroxylabda-8(17),13(14)-dien-16,15-olide. Interestingly, all four were reported with potent antiproliferative activities against various human cancer cell lines^31^.

To our knowledge, the present study qualifies to be the first report on the anticancer activity of *C. mutabilis* rhizome extract (CMRP) as well as that of the labdane diterpenoid, Cm epoxide, isolated from it. A detailed, comparative analysis of their anticancer activities, employing human cancer cell lines with normal human lymphocytes (hPBLs) and erythrocytes as controls, were demonstrative of their therapeutic potential. Interestingly, notwithstanding the potent cytotoxic, antiproliferative and anti-clonogenic activities against several human cancer cell types, both CMRP and Cm epoxide failed to show any adverse effects on normal cells. Both the extract and the compound were equally capable of selective cell killing via induction of apoptosis. Cancer cells exposed to CMRP or Cm epoxide were found to exhibit apoptotic hallmarks such as membrane blebbing, nuclear chromatin condensation, cellular/nuclear fragmentation^32^, phosphatidylserine externalization, increased intracellular ROS and Ca^2+^ levels, loss of mitochondrial membrane potential^17–20^, genotoxicity and DNA laddering^33^. Cell cycle analysis also revealed the presence of the apoptotic sub-G1 phase cell population, with fractional DNA content^34^. Transcript profiling by RT-qPCR provided additional molecular evidence confirming apoptosis induction by way of upregulation of caspases 3, -8 and -9, pro-apoptotic BAX and downregulation of anti-apoptotic Bcl-2 and IAPs such as survivin and XIAP gene expression^35^. Additionally, transcripts of genes related to pathways regulating cell survival and migration such as mTOR, c-Myc, COX 2, NF-κB (p65), *bcr/abl*, *WT1*, TGF-β1 and MMP-9^35,36^ were also found to be downregulated, excepting PTEN in cancer cells exposed to CMRP. Further, western blot analysis of CMRP-treated cancer cells also bore evidence of apoptosis related caspase 3, -7, -8, -9 activation, PARP cleavage and downregulation of Bcl-2^37^. Additional downregulation of cell cycle related cyclin B1 and chk1 gene expressions were also observed. The upregulation of γ-H2AX expression, indicative of DNA damage, lent further credence to the genotoxicity observed by way of DNA strand breaks seen as comet tails and DNA laddering due to internucleosomal cleavage^38^. Likewise, scratch and transwell invasion assays also proved CMRP-induced inhibition of adherent HCT116 cancer cell migration and invasion^39^. Assessment of acute toxicity using Swiss albino mice model also attested to the pharmacological safety of CMRP and Cm epoxide, at the tested dosages, as evidenced by the absence of deviations in hematological parameters, levels of key liver enzymes and histopathological assessment of liver tissue. It may be pertinent to note here that exposure of normal human lymphocytes and erythrocytes to CMRP or Cm epoxide, at cancer cell killing concentrations, did not produce any evidence of cellular damage.

In addition to SF20, analysis of the other two bioactive sub-fractions of CMRP, SF27 and -32 also revealed the presence of major compounds credited with various bioactivities including anticancer potential. These compounds included (1) 13-apo-beta-carotenone (an eccentric cleavage product of β-carotene)^40^, (2) trilaurin, fatty acid derivative recently used in lipid nanoparticle formulation to deliver lipophilic anticancer agents such as curcumin, docetaxel, paclitaxel more effectively for cancer cure^41^, (3) borneol, a simple bicyclic monoterpene, reported with chemosensitization potential^42^, (4) γ-sitosterol (phytosterol) which reportedly induces DNA damage, cell cycle arrest and apoptosis in various cancer cell lines^43^ and (5) β-amyrin, reported to possess antitumour activity against liver carcinoma, Hep-G2 cells, induce apoptosis, disrupt cell cycle regulation and activate JNK and p38 signaling pathways^44^. Notably, SF27 and SF32 displayed potent cytotoxicity against cancer cells with IC_50_ values < 30 µg/mL.

To sum up, it is clear from the foregoing account that in addition to Cm epoxide, CMRP also harbours compounds such as 13-apo-beta-carotenone, trilaurin, borneol, γ-sitosterol and β-amyrin. This potent mixture of bioactive components, was observed to be apoptogenic via both intrinsic and extrinsic pathways^37^. RT-qPCR analysis also showed their influence on transcription of various genes involved in cell survival/migration pathways. Being a mixture bioactive compounds, CMRP extract can potentially target multiple cellular pathways, impact physicochemical properties such as solubility thereby affecting bioavailability, antagonize development of drug resistance and reduce or neutralize the toxic/adverse effects of individual component in it^45^. From this perspective, it is interesting to note that, herbal formulations are in fact an integral part of traditional Chinese, Indian (Ayurveda) and western medicine. There are several reports on the anticancer effects of phytopharmaceuticals administered alone or in combination^45,46^.

To conclude, the present study demonstrates for the first time the anticancer potential of *C. mutabilis*, an endemic Zingiberaceae plant confined to Western Ghats of the Indian State of Kerala and that the petroleum ether extract of this plant rhizome (CMRP) is a bioresource of multi-potent bioactive compounds. This study also reports for the first time on successful isolation, identification and demonstration of apoptogenic effects of a labdane diterpenoid, Cm epoxide from CMRP. Further extensions of the present study, in terms of various dosage regimens, combination treatments with other anticancer agents and those involving advanced tumour models of human cancers, are necessary prior to advancement of these agents with promising therapeutic potential into clinical trials.

## Methods

### Plant material and extract preparation

*Curcuma mutabilis* was collected from the Nilambur forest (Lattitude–11.2806791°N and longitude–76.1987534°E), Malappuram district of Kerala state, India. The plants were authenticated by Prof. M. Sabu, Department of Botany, University of Calicut, and a voucher specimen (Accession No. 6856 CALI) was deposited in the Calicut University Herbarium. The collected plant rhizomes were washed well with running water, cut into small pieces, shade-dried, powdered using a grinder and then were subjected to serial extractions with organic solvents of increasing polarities such as petroleum ether, chloroform, acetone and methanol by gentle mixing on a gyroshaker for 24 h. The extracts were filtered through Whatman No. 1 filter paper and the filtrates evaporated to dryness. The extract residues were then dissolved in dimethylsulphoxide (DMSO) to prepare a stock solution which was stored at − 20 °C until use.

### Chemicals and reagents

Cell culture media components such as DMEM, RPMI-1640 medium, Ham’s F12 K medium, penicillin, streptomycin, Hi-Sep Lymphocyte separation medium, HiKaryo XL RPMI medium, MTT, acridine orange, BSA, skimmed milk, prestained protein marker and tween 20 were purchased from HiMedia Laboratories, Mumbai, India. DMSO, trypan blue, Hoechst 33258, DCFH-DA, Rhodamine-123, Tris base, TRI reagent, Oligo(dT)-18 mer primers, Protease/phosphatase Inhibitor Cocktail and KBr were purchased from Sigma-Aldrich, USA. Fetal bovine serum, Dulbecco’s Phosphate Buffered Saline (DPBS), Trypsin–EDTA, Alexa Fluor 488 Annexin V/Dead Cell Apoptosis Kit, Fluo-3 AM, propidium iodide and Geltrex LDEV-Free Reduced Growth Factor Basement Membrane Matrix were purchased from Thermo Fisher Scientific, USA. M-MuLV reverse transcriptase enzyme, RNase A, dNTP mix and agarose were purchased from Genei Laboratories Pvt. Ltd, Bangalore, India. Triton X-100, EDTA, glycine, ethidium bromide, BCIP, NBT, silica gel for column chromatography (60–120 meshes), vanillin, sulphuric acid and solvents such as petroleum ether, chloroform, acetone, methanol (all HPLC grade) were procured from Sisco Research Laboratories Pvt. Ltd., Mumbai, India. Antibodies were procured from Cell Signaling Technology, Inc., USA and Sigma-Aldrich, USA. Primers sets were purchased from Eurofins Genomics India, Bangalore, India.

### Cell lines and cultures

The cell lines used in the present study were chronic myelogenous leukemia—K562, acute promyelocytic leukemia—HL60, colorectal carcinoma—HCT116, hepatocellular carcinoma—Hep G2, non-small lung alveolar carcinoma—A549, prostate cancer—PC3, breast cancers—MCF7 and MDA-MB-231, embryonic kidney—HEK293T, purchased from the National Center for Cell Science (Pune), India. K562 and HL60 were maintained and propagated in RPMI 1640 medium, whilst HCT116, HepG2, MCF7, MDA-MB-231 and HEK293T were cultured in DMEM; A549 and PC3 cells were cultured in Ham’s F12K medium. All media were supplemented with 10% (v/v) fetal bovine serum, streptomycin (100 μg/mL) and penicillin (100 U/mL). Cell cultures were maintained at 37 °C in a humidified atmosphere of 5% CO_2_. hPBL cells were separated from whole blood collected from healthy donors and cultured in HiKaryo XL RPMI media according to manufacturer’s protocol. Briefly, the isolation and culture of hPBLs was carried out as follows: 5.0 mL of human blood was mixed with an equal volume of phosphate buffered saline (PBS) and then carefully overlaid on 6.0 mL of Hi-Sep Lymphocyte separation medium in a centrifuge tube and spun at 400 × *g* for 30 min at room temperature. The buffy coat obtained at the fluid interface, aspired using a sterile Pasteur pipette, was then washed twice with PBS. Finally, cells were seeded at a density of 1 × 10^5^ cells/mL into 5.0 mL HiKaryo XL RPMI medium and incubated at 37 °C for 48 h for growth.

### Ethical statement

The blood samples used for hemolysis assay and isolation of lymphocytes for lymphocyte culture and MTT assay was willingly self-donated by the first author (Soumya T.) and the second author (Lakshmipriya T.) of the manuscript submitted. It may be noted that according to the Indian Council for Medical Research, New Delhi, India (National ethical guidelines for biomedical and health research involving human participants) the ethical approval for use of self-donated blood by the researcher was not deemed to be necessary. According to this guideline, proposals which present less than minimal risks are exempted from the ethical review process.

### Cytotoxicity assay

For cytotoxicity evaluation, all cancer and normal cell types were exposed to varying concentrations (1–100 μg/mL) of different *C. mutabilis* rhizome extracts or column sub-fractions from CMRP, prepared in DMSO and incubated for 24 h, followed by MTT assay as previously described^47^.

### Trypan blue dye exclusion assay

Cell viability of extract(s) / Cm epoxide-treated cancer cells was also determined using trypan blue dye exclusion assay as previously described^48^. Briefly, cells (1 × 10^5^cells/mL) were incubated with varying concentrations of individual extract(s) or the compound for 24 h. The cells were harvested, washed with PBS (pH-7.4), stained with 0.4% trypan blue solution and counted using a haemocytometer. The result was expressed as percentage dead cells relative to control.

### Clonogenic assay

Cells treated with CMRP or Cm epoxide for 24 h were washed with PBS and counted. Aliquots of 500 cells were then plated onto a 6-well dish containing complete medium in the case of HCT116; for K562, a cell suspension in RPMI medium containing 0.3% low melting agar was used. Following 10 days of incubation, the colonies were stained with crystal violet (0.5%, w/v) and counted manually^49^.

### Analysis of alterations in cell morphology

Cytomorphological changes induced by CMRP or Cm epoxide, if any, in cancer cell lines HCT116, K562 and MDA-MB-231 were observed and photographed using light (phase-contrast), fluorescence and scanning electron (CARL-Zeiss Gemini 300 SEM) microscopic techniques.

### Nuclear morphological analysis by Hoechst 33258 staining

The control and treated cells were collected, washed twice with PBS, re-suspended in Hoechst 33258 staining solution (1 mg/mL) and incubated for 10 min at room temperature in the dark. The cells were then observed for changes in nuclear morphology under a fluorescence microscope.

### Acridine orange (AO)—ethidium bromide (EB) dual staining

The control and treated cells were harvested, washed with cold PBS (pH—7.4) and adjusted to a cell density of 1 × 10^5^ cells/mL using PBS. The AO—EB solution (1:1, v/v) was added to the cell suspension at a final concentration of 100 μg/mL prior to observation under a fluorescence microscope.

### Assessment of phosphatidylserine (PS) externalization

Externalization of PS in the treated cancer cells was evaluated by staining with Alexa Fluor 488—annexin V (Apoptosis kit, Invitrogen, USA) in accordance with the manufacturer’s instructions. In addition to fluorescence microscopy, the intensity of cellular fluorescence was also measured by flow cytometry on a BD FACS ARIA II machine with BD FACS Diva software 6.1.3.

### Assessment of intracellular reactive oxygen species (ROS)

Cellular ROS levels of treated cancer cells were studied by using the cell permeable fluorescent probe, 2′,7′ dichlorofluorescin diacetate (DCFH-DA)^18^. Briefly, the control and treated cells were stained with 10 μM DCFH-DA for 30 min at 37 °C, washed twice with PBS and observed under fluorescence microscope. The quantification of ROS was carried out using flow cytometry as mentioned earlier.

### Assessment of intracellular Ca^2+^ concentration

Alterations in the intracellular Ca^2+^ levels were determined by deploying a fluorescent dye, Fluo-3 AM^19^. The control and treated cells were washed twice with PBS, following incubation with 5 μM Fluo-3 AM for 30 min at 37 °C. The cells were then washed and subjected to fluorescence microscopy and flow cytometric analysis.

### Assessment of mitochondrial membrane potential (ΔψM)

Mitochondrial membrane potential of control and treated cells were determined by staining with the lipophilic cationic dye, rhodamine 123 (5 μg/mL) for 30 min at 37°C^20^. Cells were then washed with PBS, centrifuged, resuspended in PBS, mounted on slides for fluorescence microscopy. Flow cytometric analysis of fluorescence due to rhodamine 123 within the cells was analysed as described earlier.

### Cell cycle analysis

Flow cytometric assessment of cell cycle distribution was carried out by staining cells with DNA intercalating dye, propidium iodide (PI). Based on DNA content, the percentage of cells distributed within the sub-groups of the total cell population representing sub-G0/G1, G0/G1, S and G2/M phases of cell cycle were quantified on a BD FACS-ARIA II cytometer using BD FACS Diva software version 6.1.3. Briefly, control and treated cells were separately harvested, washed twice with PBS, fixed in 70% ice cold ethanol and preserved at − 20 °C until use. The cells were stained with 20 µg/mL PI solution containing 200 µg/mL RNase A (DNase free) for 30 min prior to data acquisition and analysis.

### Genotoxicity evaluation by comet assay

The comet assay was used to detect DNA strand breaks within single cells subjected to gel electrophoresis under alkaline conditions. Genotoxic evaluation of the CMRP extract was carried out essentially as previously described^50^.

### DNA fragmentation assay

The control and treated cells were washed with PBS, resuspended in lysis buffer (20 mM Tris; pH 8.0, 10 mM EDTA and 0.2% Triton X-100) and incubated for 15 min at room temperature. DNA was then isolated from this cell lysates by standard procedures as previously described^33^, electrophoresed on 1.8% agarose gel and observed under gel documentation system for DNA fragmentation.

### RNA isolation and RT-qPCR analysis

Total cellular RNA from the control and treated cells were isolated using TRI Reagent in accordance with the manufacturer’s protocol. The RNA samples were treated with RQ1 RNase-free DNase (Promega Coporation, USA) to digest any residual chromosomal DNA and then quantified using UV spectrophotometer (Eppendorf BioSpectrometer basic, Eppendorf AG, Germany). For first strand cDNA synthesis, initially 5 μL of reaction containing 2 μg of total RNA, 1 μL of 10 mM dNTP mix and 1 μL of 100 μM oligo dT primer were incubated at 65 °C for 10 min, spun briefly and placed in ice. To this, 2 μL of 5 × M-MuLV reverse transcriptase buffer, 0.25 μL of 20 U/μL M-MuLV Reverse transcriptase, 0.25 μL of Ribolock RNase Inhibitor (Thermo Fisher Scientific, USA) were added and made up to 10 μL with sterile nuclease free water. The reaction mixture was incubated at 37 °C for 60 min and then heated to 95 °C for 10 min and stored at − 20 °C until use. Later, 2.0 µL of the cDNA, along with SYBR Premix Ex Taq (Tli RNaseH Plus) (TaKaRa Bio Inc., Japan) and 0.25 µM of individual primer sets (listed in Supplementary Table S6) were added to a 10 µL reaction subjected to thermo-cycling in an Illumina Eco Real Time system (USA) following standard protocols. Data were analysed using the delta-delta Ct method and plotted as fold change versus control^51^.

### Immunoblotting

SDS-PAGE and western blotting were performed essentially as described previously^47^. The control and treated cells were harvested, washed with ice-cold PBS and lysed in RIPA buffer containing freshly added protease/phosphatase inhibitors. Following protein estimation of the cell lysates using Bradford’s method, aliquots of samples containing equivalent amounts (50 μg) of proteins were subjected SDS-PAGE profiling on a 4–12% gradient gel. The fractionated polypeptides were blotted onto Immobilon PVDF membrane (Merck Life Sciences Pvt. Ltd., India) blocked with 5% (*w/v*) skimmed milk prepared in TBST (TBS with 0.1% Tween 20) for 1 h at room temperature. Immunostaining was carried out overnight at 4 °C separately with mouse/rabbit-derived primary antibodies (dilution 1:1000) against β-actin, caspase -3, -7, -8, -9, PARP, Bcl-2, phospho-H2AX, cyclin B1 and Chk1. The membranes were then washed in TBST and incubated for 1 h at room temperature with either anti-mouse or anti-rabbit ALP or HRP-conjugated secondary antibodies (1:2000). Following another wash with TBST, the blots were exposed to BCIP/NBT or chemiluminescene methods to visualize the immunostained polypeptides.

### Bioassay guided isolation of active fractions and compounds

Among the various extracts tested, the petroleum ether extract of *C. mutabilis* rhizome (CMRP) which displayed potent antiproliferative activity, was subjected to further analysis of bioactive fraction/compound(s). Column chromatography of CMRP was carried out using a glass column (300 × 18 mm) of silica gel 60 (60–120 meshes). The gel was mixed well with hexane to degas and then packed into the column. The sample (CMRP—2.5 g) was mixed with a small amount of silica gel applied to the packed column without trapping any air bubbles. Using individual solvents or combinations thereof of increasing polarities, fractions were eluted using hexane (100, 100 mL), hexane : chloroform (90:10, 70:30, 50:50, 30:70 and 10:90, 100 mL), chloroform (100, 100 mL), chloroform : acetone (80:20 and 20:80, 100 mL), acetone: methanol (50:50, 100 mL) and methanol (100, 100 mL). The sub-fractions obtained as of 20 mL aliquots were analysed using TLC followed by assessment of their anticancer activities. Depending upon lowest IC_50_ values and separation pattern of bands observed in TLC, three fractions, SF20, -27 and -32 were chosen for analysis of compounds present therein.

The sub-fractions were then analysed on aluminium backed, pre-coated, silica gel 60 F_254_ TLC plates (Merck, Germany) using toluene: ethyl acetate (7:3, *v/v*) solvent system for SF20 and toluene: ethyl acetate: methanol (18:1:1, *v/v*) for SF27 and -32, followed by derivatization with vanillin-sulphuric acid reagent. SF20 was further characterized with UV–visible spectroscopy (Perkin-Elmer spectrophotometer) and HPLC (Shimadzu Prominance system) under standard conditions. The mass of the purified compound was analysed by HPLC-HRMS system (Thermo scientific Q Exactive orbitrap mass spectrometer fitted with HypersilTM C18-column). The mobile phase consisted of a gradient of 97% methanol and 3% water with 0.1% formic acid for 5 min at a flow rate of 150 µL/min. The sample injection volume was 2 µL and detection was carried out at 235 nm. Electrospray ionization source was operated in both positive and negative mode with an ion spray voltage of 3KV. Oven temperature was set to 30 °C and *m/z* scan range was kept between 100−2000 Da.

### Identification of compounds

For FTIR analysis, 1 mg of SF20 was finely ground with about 100 mg of KBr, pressed into a thin disc by using hydraulic press and then connected to a vacuum chamber to dehydrate the sample. FTIR spectroscopy of this KBr disc was then carried out using Jasco 4100 IR spectrometer within the frequencies ranging from 4000–400 cm^−1^. NMR spectra were acquired using a Bruker Avance NMR spectrometer equipped with a 5 mm inverse-configuration probe of triple-axis-gradient capability with a field-strength of 14.1 T operating at 600.1 and 150.9 MHz for ^1^H and ^13^C nuclei, respectively. Spectra were acquired at 25 °C in samples containing CDCl_3_. The chemical shifts of ^1^H and ^13^C nuclei are reported relative to TMS (δ = 0 ppm for both ^1^H and ^13^C). The chemical shifts of ^1^H nuclei are reported to three decimal places when the multiplet was amenable to first-order analysis or to two decimal places when the multiplet was beyond such interpretation; for overlapped signals, chemical shifts were taken from 2D NMR spectra and are reported to three decimal places to distinguish them. General NMR experimental and acquisition details for 1D ^1^H, ^13^C, and DEPT observation and standard gradient-selected 2D COSY, HSQC, HMBC,and NOESY spectra and routine chemical shift assignment using 2D NMR have been previously described^52–54^.

### GC–MS analysis of SF27 and -32

GC–MS analysis of sub-fractions was carried out separately in an Agilent gas chromatography 6850 Network GC system (Agilent technologies, USA), fitted with a HP-5MS capillary column (30 m length × 0.25 mm ID × 0.25 μm film thickness, interfaced with an Agilent 5975C VLMSD with triple axis mass detector). Initially, the oven was maintained at 60 °C for 1 min and the temperature was then gradually increased to 250 °C at a rate of 5 °C /min. Injector temperature was kept at 280 °C. Helium was used as a carrier gas, adjusted to column velocity flow of 1 mL/min with a split ratio of 10:1 and a split flow at 10 mL/min. The sample (1 μL) was injected into the system. Identification of components was based on comparison with mass spectral fragmentation patterns of those stored in the MS library [NIST databases—NIST08 and NIST08.L (National Institute of Standards and Technology, USA)].

### In vivo toxicological studies

All animal experiments were conducted in accordance with the CPCSEA guidelines and all experimental protocols were approved by ethical committee for animal care, Kannur University as stipulated by Indian National Law on animal care and use. The experimental design was approved by the Institutional animal ethics committee (KULS/IAEC/2019/28), Kannur University. Swiss albino mice, 6–8 weeks old and weighing approximately 18–22 g were maintained in well-ventilated cages under standard conditions of room temperature, pressure, and humidity and fed with mice standard diet (VRK Nutritional solutions, Pune, India) and water ad libitum, at 12 h light/dark cycle. To determine acute toxicity, a single dose of CMRP (50 and 200 mg/kg body weight) and Cm epoxide (25 and 100 mg/kg body weight) were administered orally to groups of 6 animals each. The mice were then observed for the next 7 days for any gross behavioural change and death. Following euthanization on the 8th day, hematological parameters (Hb content, RBC and WBC counts) and liver function tests were carried out by SI methods, along with histopathological analysis of liver tissue by H-E staining.

### Molecular docking

Molecular docking was carried out using Schrodinger maestro Glide software as manufacture instructions. Native 3D protein structures of EGFR, HER2, VEGFR, B-RAF, ABL kinase, CDK2, HDAC, PI3K and AKT were downloaded from PDB database followed by refining for Glide docking using protein preparation wizard tool. Known inhibitors of these proteins were chosen from PubChem database to enable a comparison with that of Cm epoxide’s docking efficiency. Ligand docking was carried out using prepared ligand(s) and receptor-grid-generated protein in XP (extra precision) mode. Docking scores representing the sum of all interactions between the amino acid residues at the binding site and the ligand molecule as well as overall binding energy of whole protein–ligand complex predicted by Molecular Mechanics/Generalized Born Surface Area (MMGBSA) scores were analysed, for choosing best targets of Cm epoxide.

### Statistical analysis

All experimental data are expressed as mean ± SD from three independent experiments. Results were analysed for significance by one-way ANOVA using SPSS software version 16.0.

## Supplementary Information

Supplementary Information.
