# Supplementary material for: Anticancer potential of rhizome extract and a labdane diterpenoid from Curcuma mutabilis plant endemic to Western Ghats of India
Source: Sci Rep. 2021 Jan 12;11:552. doi: 10.1038/s41598-020-79414-8 (PMC7803788; doi:10.1038/s41598-020-79414-8)
Supplement: Supplementary file 1 — Supplementary Information. [file 41598_2020_79414_MOESM1_ESM.pdf]

<u>**Supplementary information**</u>

Anticancer potential of rhizome extract and a labdane diterpenoid from *Curcuma mutabilis* plant endemic to Western Ghats of India

T. Soumya [1], T. Lakshmipriya [1], Karel. D. Klika [2], P. R. Jayasree [3] and P. R. Manish Kumar [1*]

[1] Department of Biotechnology, University of Calicut, Kerala, India.

[2] Molecular Structure Analysis, German Cancer Research Center (DKFZ), Heidelberg, Germany

[3] School of Health Sciences, University of Calicut, Kerala, India.

* Corresponding author - Email : prmanishkumar@uoc.ac.in
Cell no. : +91-9447760771
ORCID ID – 0000-0002-8987-8439

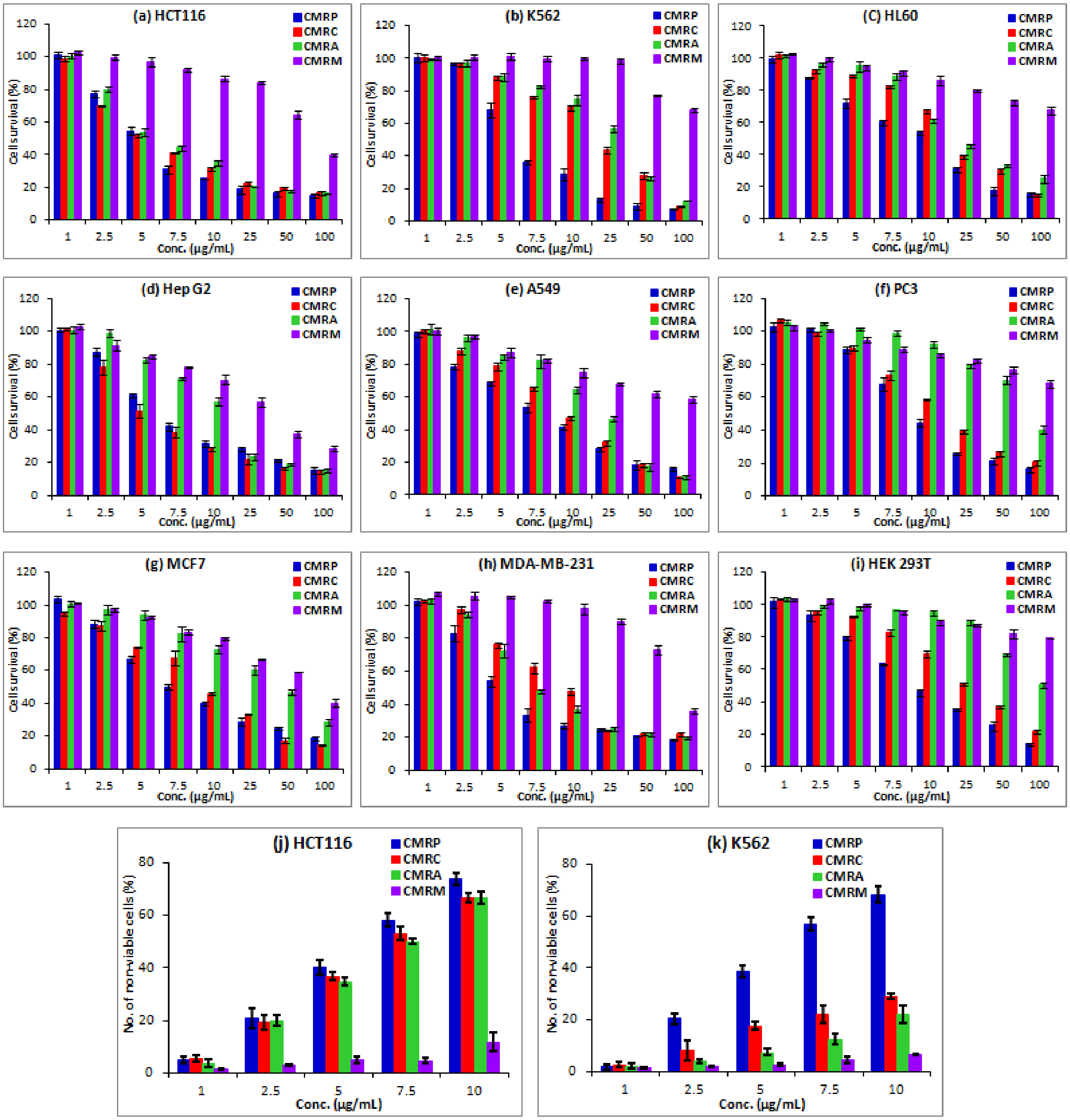

Supplementary Figure S1. Evaluation of cytotoxicity induced by *Curcuma mutabilis* rhizome (CMR) extract on human cancer cell lines. Percentage of cell survival of (a) HCT116, (b) K562, (c) HL60, (d) Hep G2, (e) A549, (f) PC3, (g) MCF7, (h) MDA-MB-231 and (i) HEK293T treated with varying concentrations of (1-100 µg/mL) organic solvent extracts from *C. mutabilis* rhizome for 24 h assessed by MTT assay. Percentage of non-viable cells in extract treated (j) HCT116 and (k) K562 cells as determined by trypan blue dye exclusion assay. Values represent mean ± SD of three independent experiments; $P < 0.05$.

Supplementary Table S1. IC_{50} values of *C. mutabilis* rhizome extracts (μg/mL) for 24 h, determined using MTT and Trypan blue dye exclusion assays.

Cell lines	IC_{50} values of *C. mutabilis* rhizome extracts (μg/mL) for 24 h							
	CMRP		CMRC		CMRA		CMRM	
	MTT	Trypan blue	MTT	Trypan blue	MTT	Trypan blue	MTT	Trypan blue
HCT116	5.4 ± 1.1	6.1 ± 1.5	5.5 ± 1.5	6.8 ± 2.4	6.0 ± 0.9	7.5 ± 1.0	79.5 ± 4.8	83.5 ± 2.2
K562	6.5 ± 1.0	6.8 ± 1.2	21.8 ± 1.7	26.8 ± 3.4	30.5 ± 2.7	33.0 ± 2.2	> 100	> 100

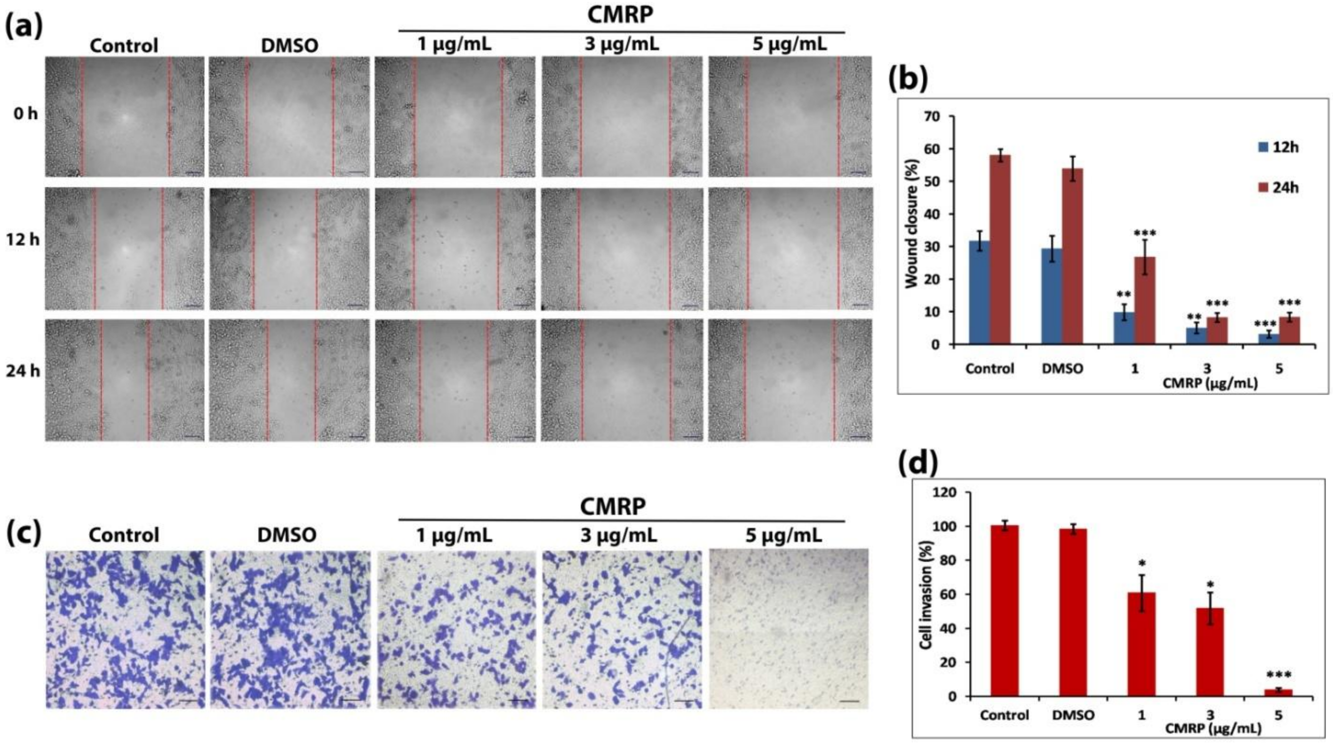

Supplementary Figure S2. Effect of CMRP on HCT116 cell migration and invasion. (a) Inhibition of HCT116 cell migration observed for 0, 12 and 24 h, following treatment with CMRP analysed by *in vitro* scratch (wound healing) assay. (b) Histogram showing percentage of wound closure at 12 and 24 h. (c) Cells invade through Geltrex®-coated transwell inserts in response to 24 h CMRP treatment. (d) Histogram showing cell invasion. Data represented as mean ± SD of three independent experiments. $^*P < 0.05$, $^{**}P < 0.01$, $^{***}P < 0.001$.

Supplementary Table S2. Solvent system profiles and cytotoxicity (IC_{50}) of 55 sub-fractions of CMRP

Sub-fractions (SF)	Solvent system	IC_{50} values (µg/mL) for 24 h	
		**HCT116**	**K562**
1 – 5	Hexane (100%)	> 100	> 100
6	Hexane:Chloroform (90 : 10)	33.5 ± 1.86	38.1 ± 1.72
7 – 10		> 100	> 100
11 – 14	**Hexane : Chloroform (70 : 30)**	> 100	> 100
**15**		**12.6 ± 1.2**	**21 ± 1.2**
**16 – 20**	**Hexane : Chloroform (50 : 50)**	**≤ 2.5**	**≤ 2.5**
**21 – 24**	**Hexane : Chloroform (30 : 70)**	**≤ 2.5**	**≤ 2.5**
**25**		**4.4 ± 0.45**	**3 ± 0.5**
**26**	**Hexane : Chloroform (10 : 90)**	**18.1 ± 0.85**	**8.9 ± 0.38**
**27**		**17.5 ± 0.75**	**6.3 ± 0.35**
**28**		48.3 ± 2.61	**4.5 ± 1.15**
29		83.6 ± 4.0	43 ± 4.5
30		62.1 ± 2.07	48.5 ± 1.32
31	**Chloroform (100%)**	40.5 ± 1.94	46.4 ± 1.29
**32**		**16 ± 1.15**	**28 ± 0.81**
**33**		45.6 ± 1.3	**17.5 ± 0.72**
34		43.8 ± 0.85	46.2 ± 2.4
35		80.4 ± 0.87	50 ± 0
36	Chloroform : Acetone (80 : 20)	> 100	50.3 ± 0.3
37		87.8 ± 3.01	84.1 ± 1.65
38		75 ± 2.46	45.9 ± 0.79
39 – 40		> 100	> 100
41 – 45	Chloroform : Acetone (20 : 80)	> 100	> 100
46 – 50	Acetone : Methanol (50 : 50)	> 100	> 100
50 -55	Methanol (100%)	> 100	> 100

Values represent mean ± SD (n=3)

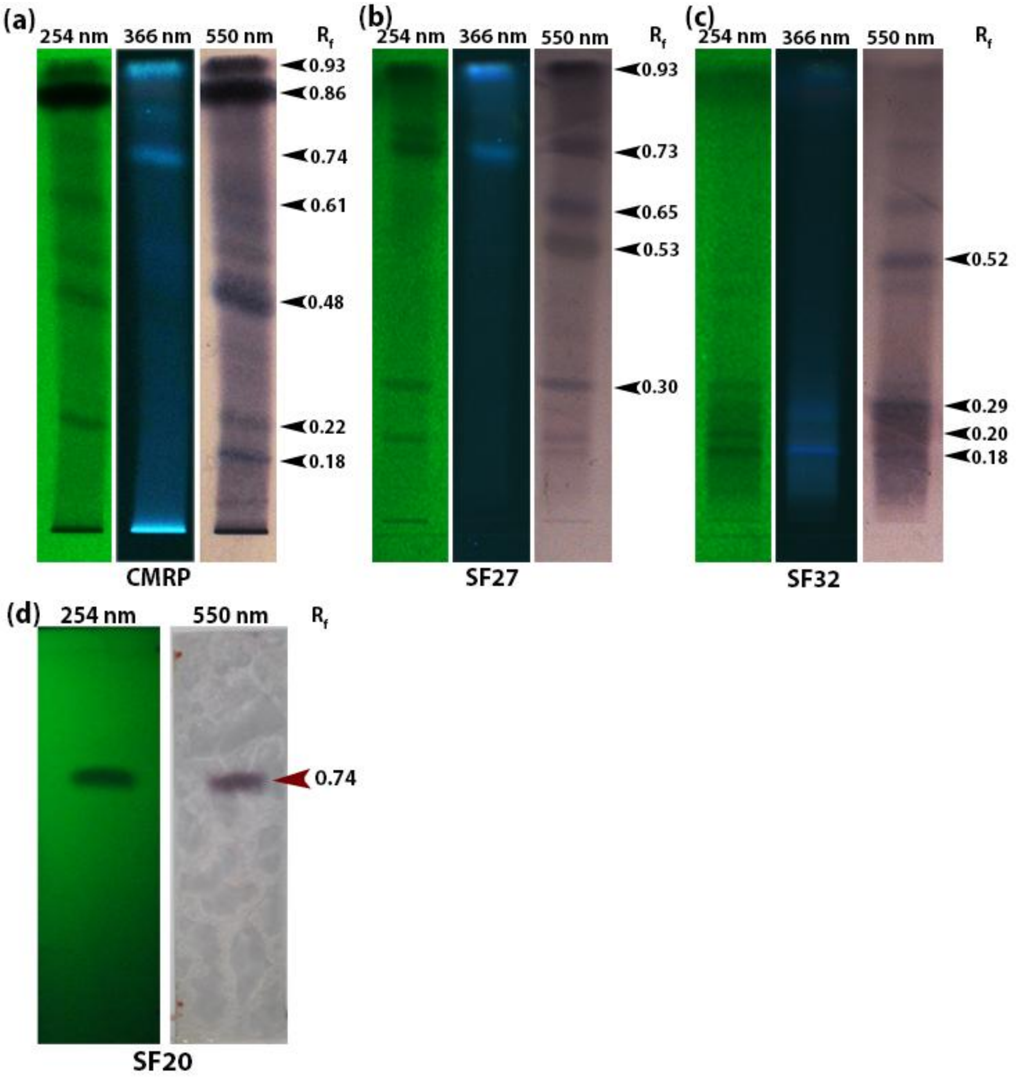

Supplementary Figure S3. TLC of CMRP, SF20, -27 and -32. (a), (b) & (c) TLC fingerprint of CMRP, SF27 and SF32 respectively, using toluene: ethyl acetate: methanol (18:1:1, *v/v*) as solvent system; visualized under UV (254 and 366 nm) and visible light (550 nm) following derivatization with anisaldehyde-sulphuric acid reagent. (d) TLC fingerprint of SF20 using toluene: ethyl acetate (7:3) solvent system, visualized under UV (254 nm) and visible light (550 nm) following derivatization with vanillin – sulphuric acid reagent. Arrowheads denote R_f values of fractionated bands.

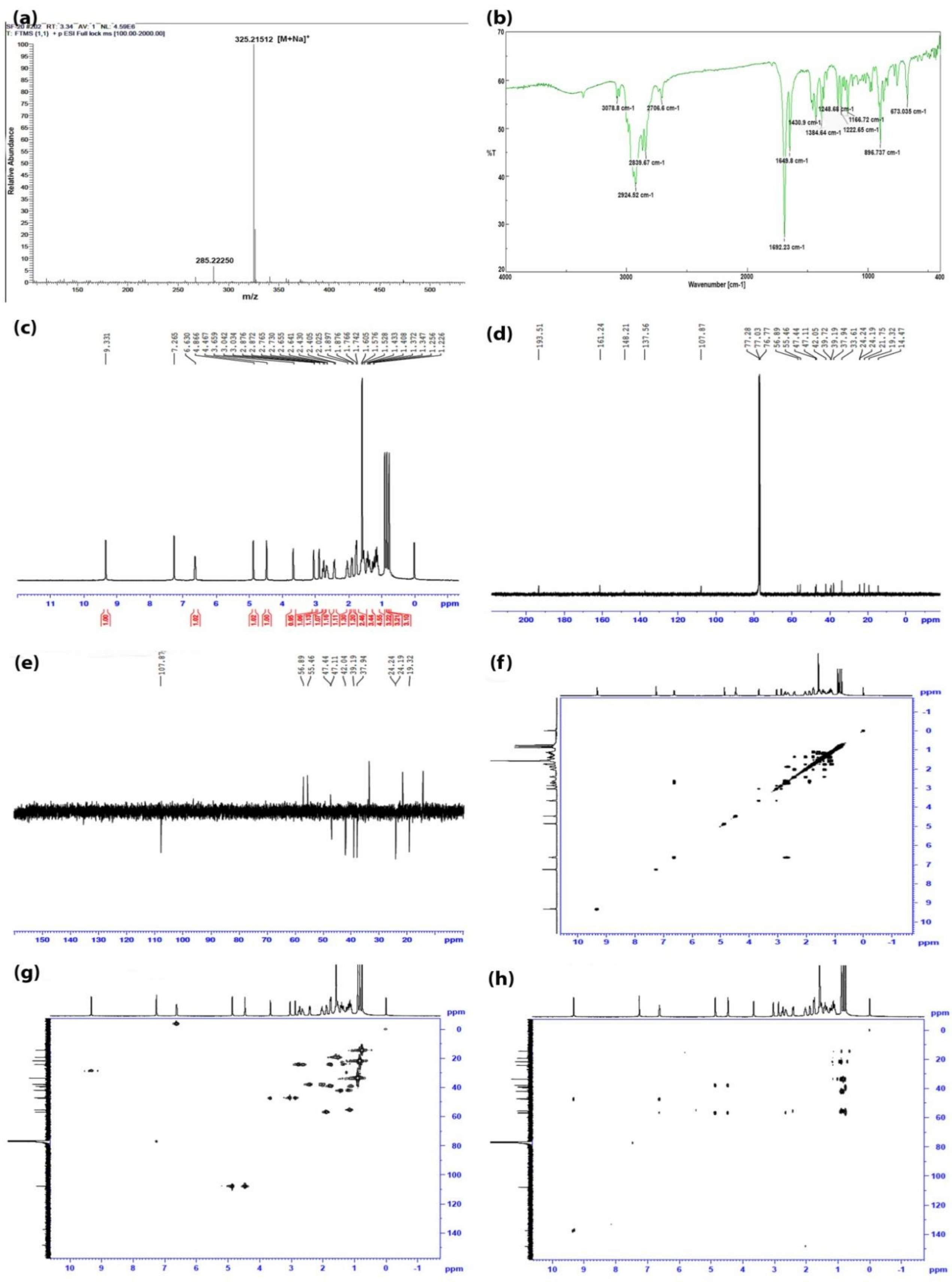

Supplementary Figure S4. HR-ESI-MS, FTIR and NMR analysis of bioactive SF20. (a) HR-ESI-MS, (b) FTIR, (c) ^{13}C NMR, (d) ^{1}H NMR, (e) DEPT135, (f) COSY, (g) HMQC and (f) HMBC spectra of SF20 from CMRP extract.

Supplementary Table S3. GC-MS analysis of chemical composition of SF27 and SF32

Column fractions	Retention Time (min)	Hit Name	MW (amu)	Peak area (%)
**SF27**	6.444	Borneol	154.136	4.00
	11.509	2,4-Di-tert-butylphenol	206.167	4.48
	14.814	1-Nonadecene	266.297	1.46
	17.609	3-Eicosene, (E)-	280.313	1.41
	19.878	Unidentified	218.152	5.59
	20.635	Octacosyl acetate	452.459	1.90
	21.621	Unidentified	142.136	7.26
	23.496	(3E,5E,7E)-6-Methyl-8-(2,6,6-trimethyl-1-cyclohexenyl)-3,5,7-octatrien-2-one	258.198	18.10
	23.693	Heptacosyl acetate	438.444	1.91
	32.358	Dodecanoic acid, 1,2,3-propanetriyl ester	638.549	10.89
**SF32**	17.428	Unidentified	236.214	3.77
	19.878	Unidentified	232.11	13.13
	20.667	Unidentified	426.386	12.23
	23.496	(3E,5E,7E)-6-Methyl-8-(2,6,6-trimethyl-1-cyclohexenyl)-3,5,7-octatrien-2-one	258.198	20.08
	24.384	Cholestane, 3,5-dichloro-6-nitro-, (3.beta.,5.alpha.,6.beta.)-	485.283	2.00
	25.535	Bicyclo[4.1.0]heptane, 7-bicyclo[4.1.0]hept-7-ylidene-	188.157	16.80
	31.059	γ-Sitosterol	414.386	2.72
	31.816	β-Amyrin	426.386	2.48

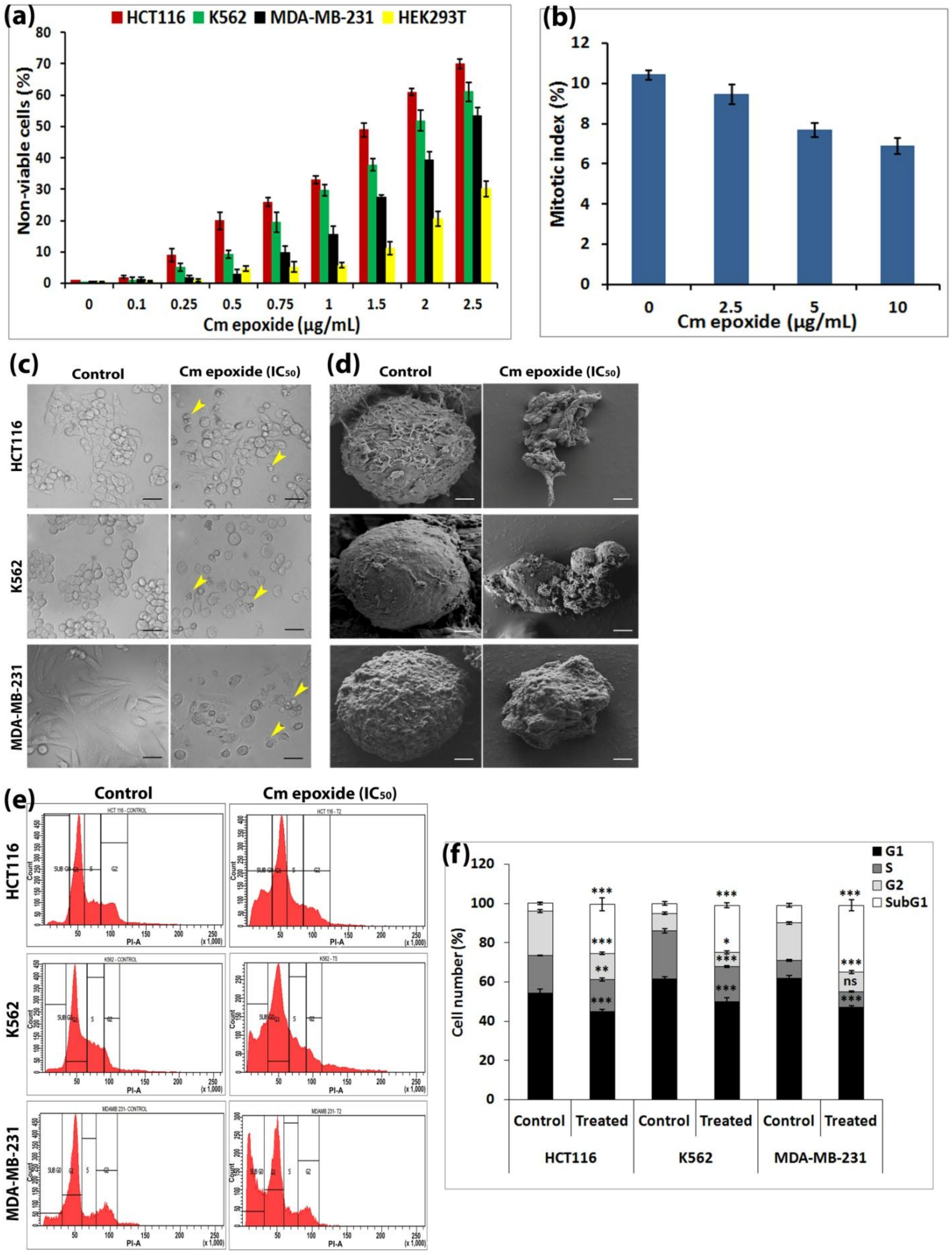

Supplementary Figure S5. Cm epoxide induces cytotoxicity and apoptosis in HCT116, K562 and MDA-MB-231 cancer cells. (a) Evaluation of viability of HCT116, K562, MDA-MB-231 and HEK293T cells following 24 h exposure to Cm epoxide by trypan blue dye exclusion assay and (b) Cytotoxicity of Cm epoxide against hPBLs represented by Mitotic index. Values represent mean ± SD of three experiments; *P* <

0.05. Cm epoxide-induced cytomorphological changes in HCT116, K562 and MDA-MB-231cells following 24 h treatment at IC_{50} concentration (c) Light microscopy images (arrow heads indicate cell shrinkage; scale bars : 10 µm) and (d) SEM images (scale bars : 2 µm). Cell cycle distribution analysis following 24 h exposure to Cm epoxide (IC_{50}) in HCT116, K562 and MDA-MB-231 cells (e) flow cytometric data representing PI stained DNA content and (f) histogram showing percentage distribution of phase-specific cells. Data represents mean ± SD of three independent experiments; ns – not significant. *$P < 0.05$, **$P < 0.01$, ***$P < 0.001$,

Supplementary Table S4. Acute toxicity evaluation: hematological parameters of CMRP and Cm epoxide treated mice

**Groups**	**Hb content (%g)**	**RBC (cells x 10^6 /mm^3)**	**WBC (cells x 10^3 /mm^3)**
**Control (5% DMSO)**	11.9 ± 0.53	7.4 ± 0.33	2.9 ± 0.37
**CMRP - 50 mg/kg**	12.0 ± 0.48	7.3 ± 0.36	2.8 ± 0.25
**CMRP – 200 mg/kg**	12.0 ± 0.57	7.4 ± 0.31	2.5 ± 0.21
**Cm epoxide – 25 mg/kg**	12.2 ± 0.39	7.2 ± 0.30	2.7 ± 0.29
**Cm epoxide – 100 mg/kg**	12.1 ± 0.69	6.9 ± 0.37	2.6 ± 0.43

Values represent mean ± SD (n=6).

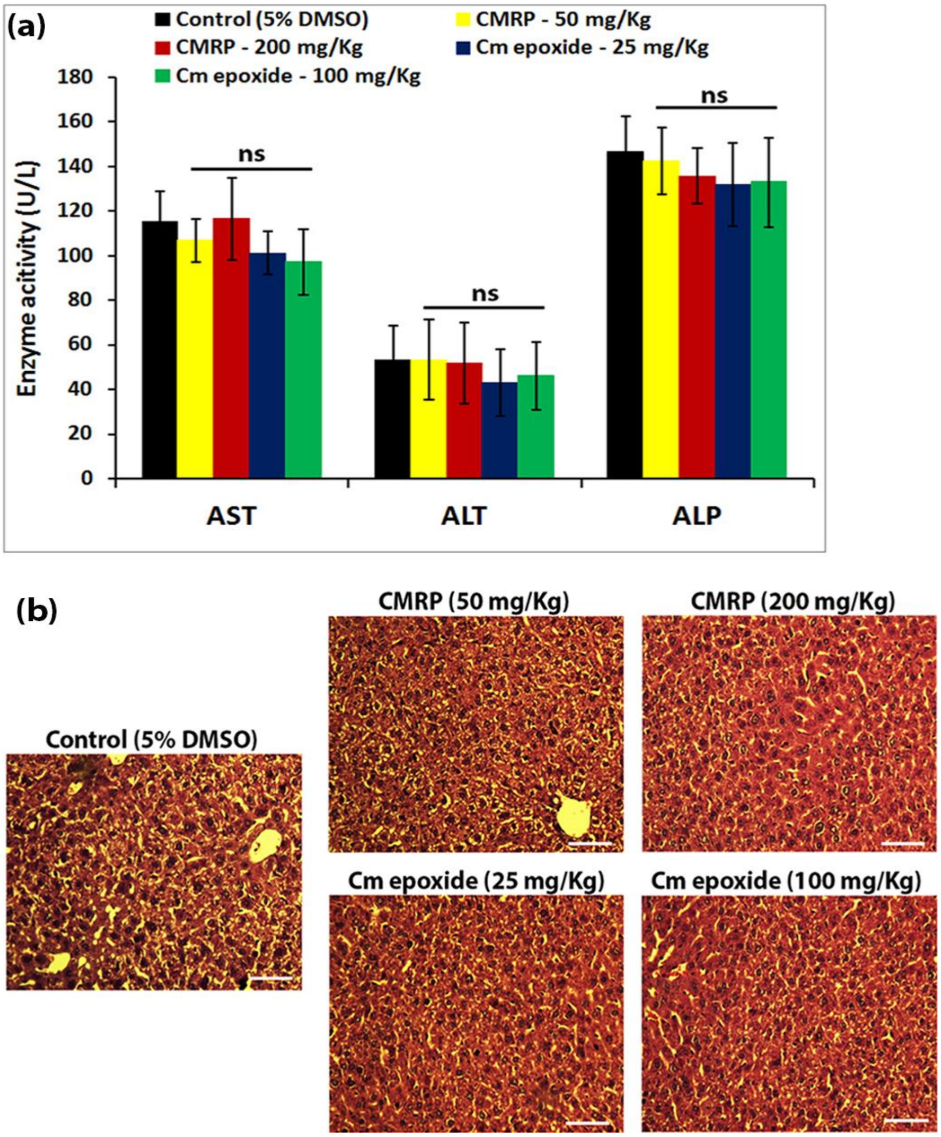

Supplementary Figure S6. CMRP and Cm epoxide found to be pharmacology safe in Swiss albino mice by acute toxicity assessment. Evaluation of hepatotoxicity by (a) liver enzyme - AST, ALT and ALP levels, Values represent mean ± SD (n=6); ns – not significant and (b) histopathology of H & E stained liver tissue sections from control and treated samples (scale bars represent 100 μm).

Supplementary Table S5. Molecular docking scores, binding energies (MMGBSA scores) and corresponding hydrogen bond interactions of the target proteins such as EGFR (PDB ID: 3POZ), B-RAF (3OG7), CDK2 (4BCP) with their specific inhibitors (ligands) and Cm epoxide.

Protein (PDB ID)	Ligand	Docking score	MMGBSA score	Number of hydrogen bonds and amino acid residues involved in H-bond (bond length in Å)
**EGFR (3POZ)**	TAK285	-14.476	-59.618	3 H-bonds, MET 793 (1.98), CYS 797 (2.11), LEU 718 (1.90)
	Gefitinib	-8.716	-45.908	2 H-bonds, LYS 245 (1.68), ASN 842 (2.21)
	Erlotinib	-4.418	-43.507	2 H-bonds, MET 793 (1.89), CYS 797 (1.75)
	**Cm epoxide**	**-6.597**	**-33.018**	**2 H-bonds, MET 793 (1.91), THR 854 (2.22)**
**B-RAF (3OG7)**	Vermurafenib	-13.695	-45.229	6 H-bonds, GLY 596 (2.16), LYS 483 (2.19), ASP 594 (2.42), ASP (2.39), GLN (2.00), CYS 532 (2.04)
	Sorafenib	-6.985	-34.816	3 H-bonds, ASP 594 (1.58), THR 529 (2.74), THR 529 (2.28)
	Dabrafenib	-4.821	-19.278	2 H-bond, SER 536 (2.09), GLN 530 (2.76)
	**Cm epoxide**	**-5.763**	**-30.684**	**1 H-bonds, CYS 532 (1.82)**
**CDK2 (4BCP)**	Roscovitine	-10.181	-38.445	3 H-bonds, LEU 83 (1.66), LEU 83 (2.35), GLU 12 (1.95)
	Flavopiridol	-9.831	-36.855	3 H- bonds, LEU 83 (1.53), LEU 83 (2.22), ASP 86 (1.79)
	Caffeine	-5.292	-23.715	1 H-bond, LEU 83 (2.08)
	**Cm epoxide**	**-5.187**	**-26.699**	**1 H-bond, LEU 83 (2.17)**

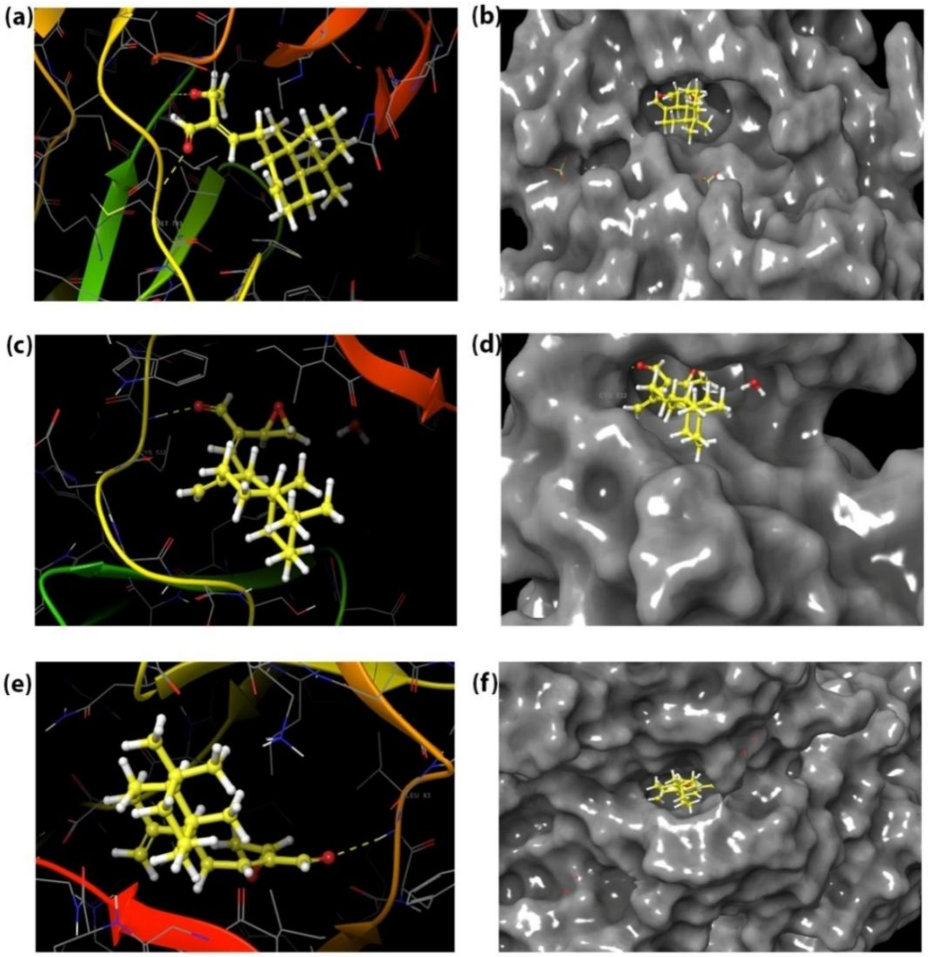

Supplementary Figure S7. Molecular docking interactions between target proteins and Cm epoxide (yellow stick) - (a, c and e) binding pose in the active site in 3D of EGFR (3POZ), B-RAF (3OG7) and CDK2 (4BCP) respectively; (b, d and f) surface representation in 3D of EGFR (3POZ), B-RAF (3OG7) and CDK2 (4BCP) respectively.

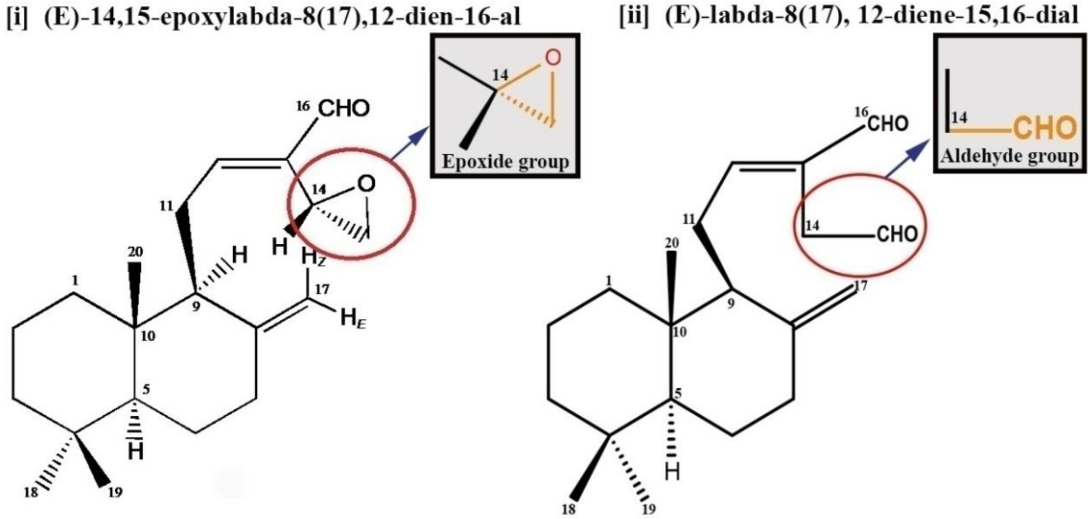

Supplementary Figure S8. Comparison of chemical structures of [i] Cm epoxide - (E)-14,15-epoxylabda-8(17),12-dien-16-al) isolated and identified from *C. mutabilis* and [ii] (E)-labda-8(17), 12-diene-15,16-dial reported from various *Alpinia* species and *C. amada.*

Supplementary Table S6. List for primer pairs used in RT - qPCR

Target gene	Orientation*	Primer sequence (5' – 3')
Caspase 3	F	TGGCATACTCCACAGCACCTGGTTA
	R	CATGGCACACAAAGCGACTGGATGAA
Caspase 8	F	CATCCAGTCACTTTGCCAGA
	R	GCATCTGTTTCCCCATGTTT
Caspase 9	F	TTCCCAGGTTTTGTTTCCTG
	R	CCTTTCACCGAAACAGCATT
PUMA	F	GACCTCAACGACAGTACGA
	R	GAGATTGTACAGGACCCTCCA
BAX	F	GCCACCAGCCTGTTTGAG
	R	CTGCCACCCAGCCACCC
Bcl-2	F	TATAAGCTGTCGCAGAGGGGCTA
	R	GTACTCAGTCATCCACAGGGCGAT
Survivin	F	ATGGGTGCCCCGACGTTG
	R	AGAGGCCTCAATCCATGG
XIAP	F	GGCCATCTGAGACACATGCAG
	R	GCATTCACTAGATCTGCAACC
mTOR	F	ATTTGATCAGGTGTGCCAGT
	R	GCTTAGGACATGGTTCATGG
PTEN	F	CAAGATGATGTTTGAAACTATTCCAATG
	R	CTTTAGCTGGCAGACCACAA
BCR/ABL	F	CTCCAGACTGTCCACAGCATTCCG
	R	CAGACCCTGAGGCTCAAAGTCAGA
WT1	F	GGCATCTGAGACCAGTGAGAA
	R	GAGAGTCAGACTTGAAAGCAGT
c-Myc	F	AAAGGCCCCCAAGGTAGTTA
	R	GCACAAGAGTTCGTTAGCTG
COX-2	F	TTCAAATGAGATTGTGGGAAAAT
	R	AGATCATCTCTGCCTGAGTATCTT
TGF-β1	F	GAGCCTGAGGCCGACTACTA
	R	GGGTTCAGGTACCGCTTCTC
TIMP 1	F	CTTCTGGCATCCTGTTGTTG
	R	GGTATAAGGTGGTCTGGTTG
MMP-2	F	GGCCCTGTCACTCCTGAGAT
	R	GGCATCCAGGTTATCGGGGA
MMP-9	F	CGGAGCACGGAGACGGGTAT
	R	TGAAGGGGAAGACGCACAGC
NF-κB	F	CACCTCAATGGCTACACAGGACCA
	R	ATCTTGAGCTCGGCAGTGTT
GAPDH	F	TCCCTGAGCTGAACGGGAAG
	R	GGAGGAGTGGGTGTCGCTGT

*F/R – forward / reverse primer

Method used for cell migration and invasion assay. HCT116 cells were seeded onto 35-mm culture dishes to obtain a confluent monolayer, which was then scratched in the middle using a sterile 200 µL micropipette tip to create an artificial gap. The dislodged floating cells were removed by a gentle wash with the growth media. Cell were then exposed to varying concentrations of the extract and the cell migratory capacity in terms of gap closure was monitored at 0, 12 and 24 h intervals by taking a series of photographs of the cells in the scratched area[1]. For invasion assay, HCT116 cells were allowed to grow to sub-confluency (75–80%) and were then serum-starved for 24 h. Following trypsinzation, the detached cells were washed with PBS, resuspended in serum-free medium and 2.5 x 10^5 cells/mL were transferred to the upper chamber of transwells (8 µm pore size) coated with 1.0

mg/mL Geltrex® matrix, along with varying concentrations of the extract. The bottom chamber was supplied with complete growth medium with 10 % FBS to serve as a chemo-attractant to induce invasion. After 24 h of incubation at 37°C, cells that had not migrated were removed from the upper face of the filters using cotton swabs. The sub-population of cells that invaded the pores of matrix into the lower surface of the transwell were fixed in methanol, stained with 0.2 % crystal violet and counted using a microscope[1,2.]

1. Zheng L, Zhang Y, Zhan Y, Liu C. *Momordica cochinchinensis* seed extracts suppress migration and invasion of human breast cancer ZR-75-30 cells via down-regulating MMP-2 and MMP-9. A*sian Pacific J. Cancer Prev.* **15**,1105–1110 (2014).

2. Zhu, P. *et al.* Inhibition of Growth and Metastasis of Colon Cancer by Delivering 5-Fluorouracil-loaded Pluronic P85 Copolymer Micelles. *Sci. Rep.* **6**, 20896; doi: 10.1038/srep20896 (2016).

Original agarose gels and western blots

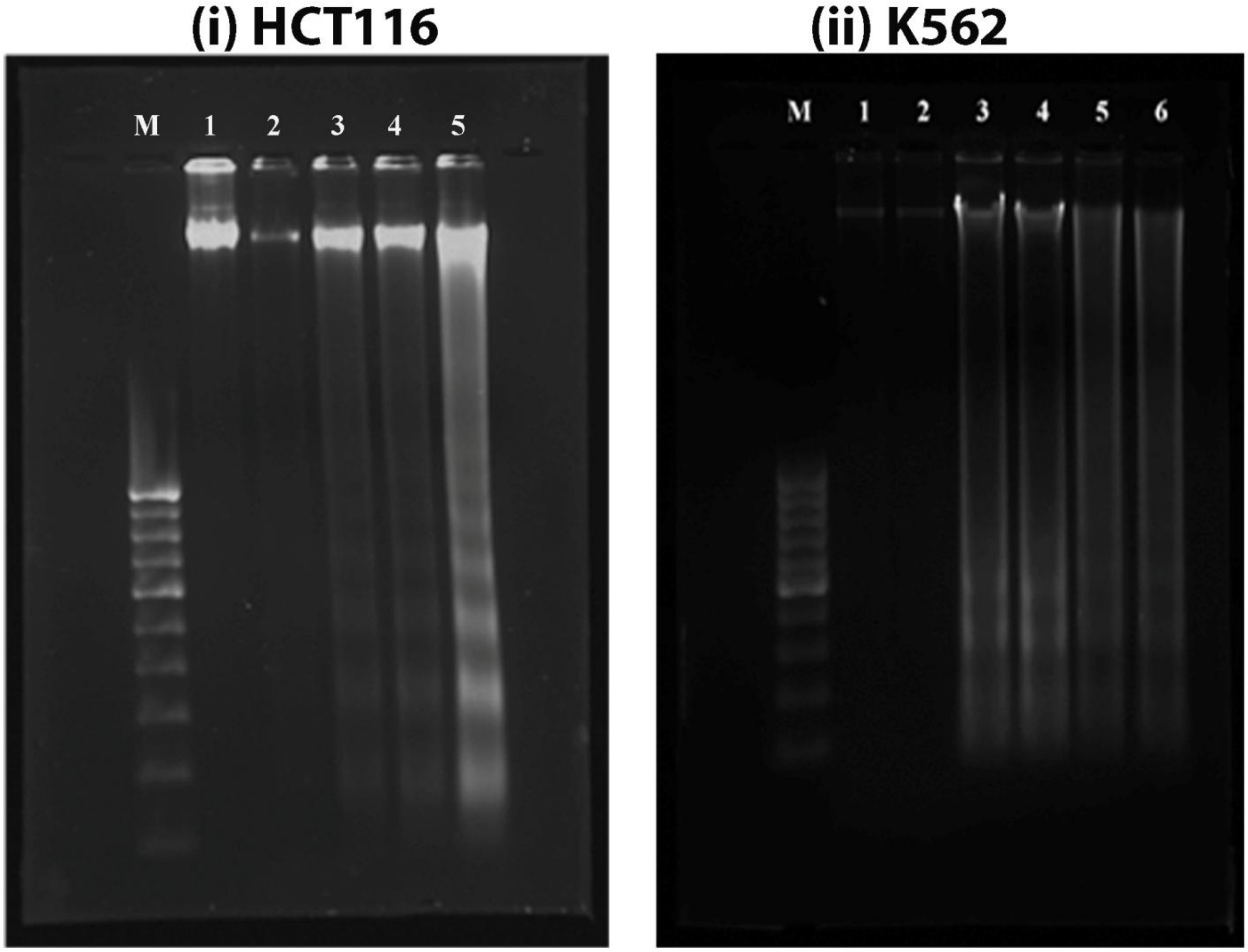

Supplementary Figure S9. Full length agarose gels displayed in Figure 7b of the main article, DNA fragmentation / laddering assay of HCT116 and K562 cells treated with CMRP for 24h. Lane M: 100bp DNA ladder, Lane 1: DNA from control cells, Lane 2: DNA from DMSO-treated cells, Lane 3: DNA from CMRP (3 µg/mL) treated cells, Lane 4: DNA from CMRP (5.5 µg/mL for HCT116 and 6.5 µg/mL for K562) treated cells, Lane 5: DNA from CMRP (12 µg/mL) treated cells.

Western blot analysis

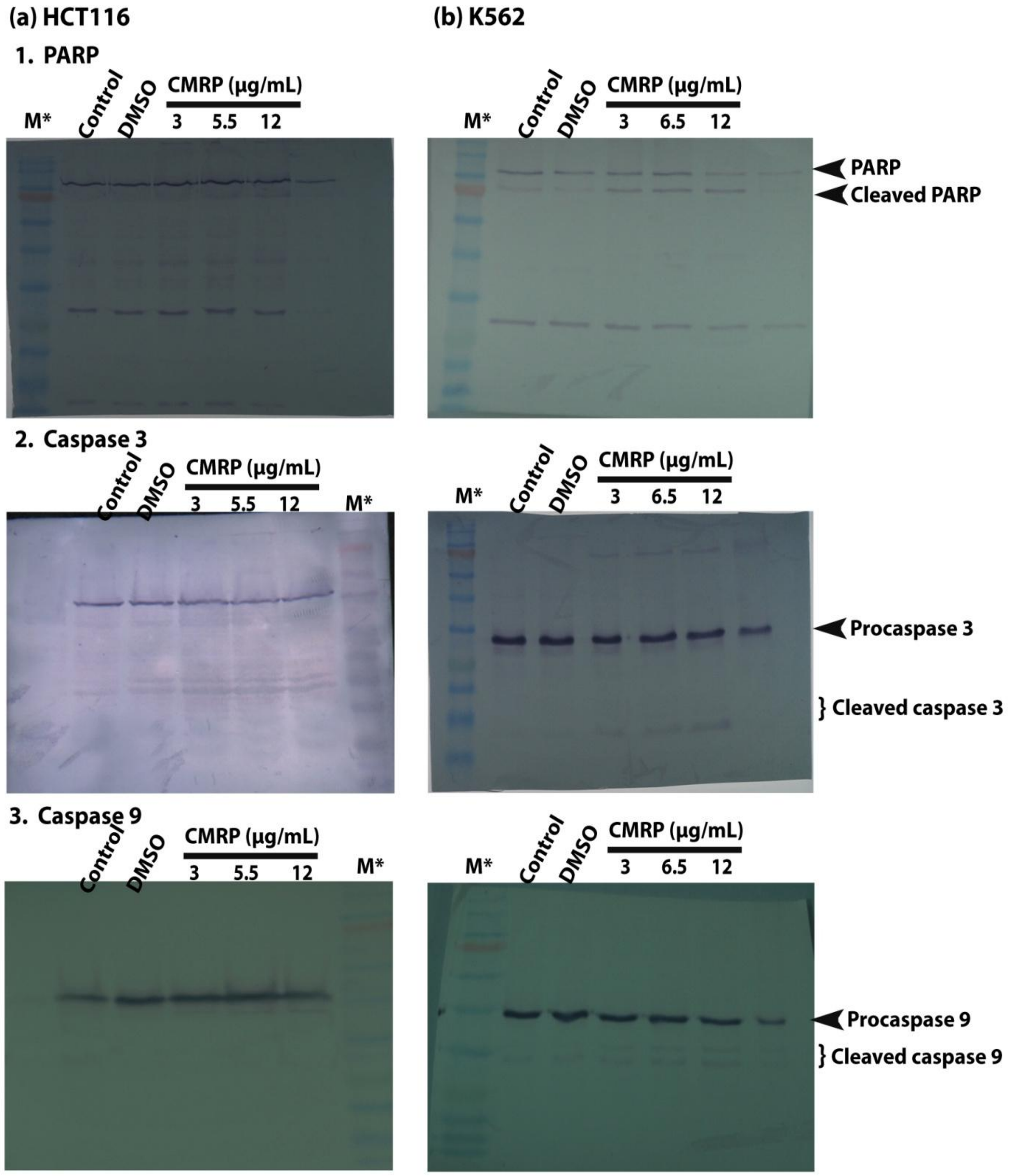

Supplementary Figure S10. Full length western blot presented in Figure 9 of the main article, Effect of CMRP on expression of apoptosis related proteins in HCT116 and K562 cancer cells analysed by western blotting using antibodies against PARP, caspase -3 and -9. Lane M* represent prestained protein marker, with 12 prestained proteins covering a wide range molecular weights from 10 to 245 kDa, **(Figure continued in next page).**

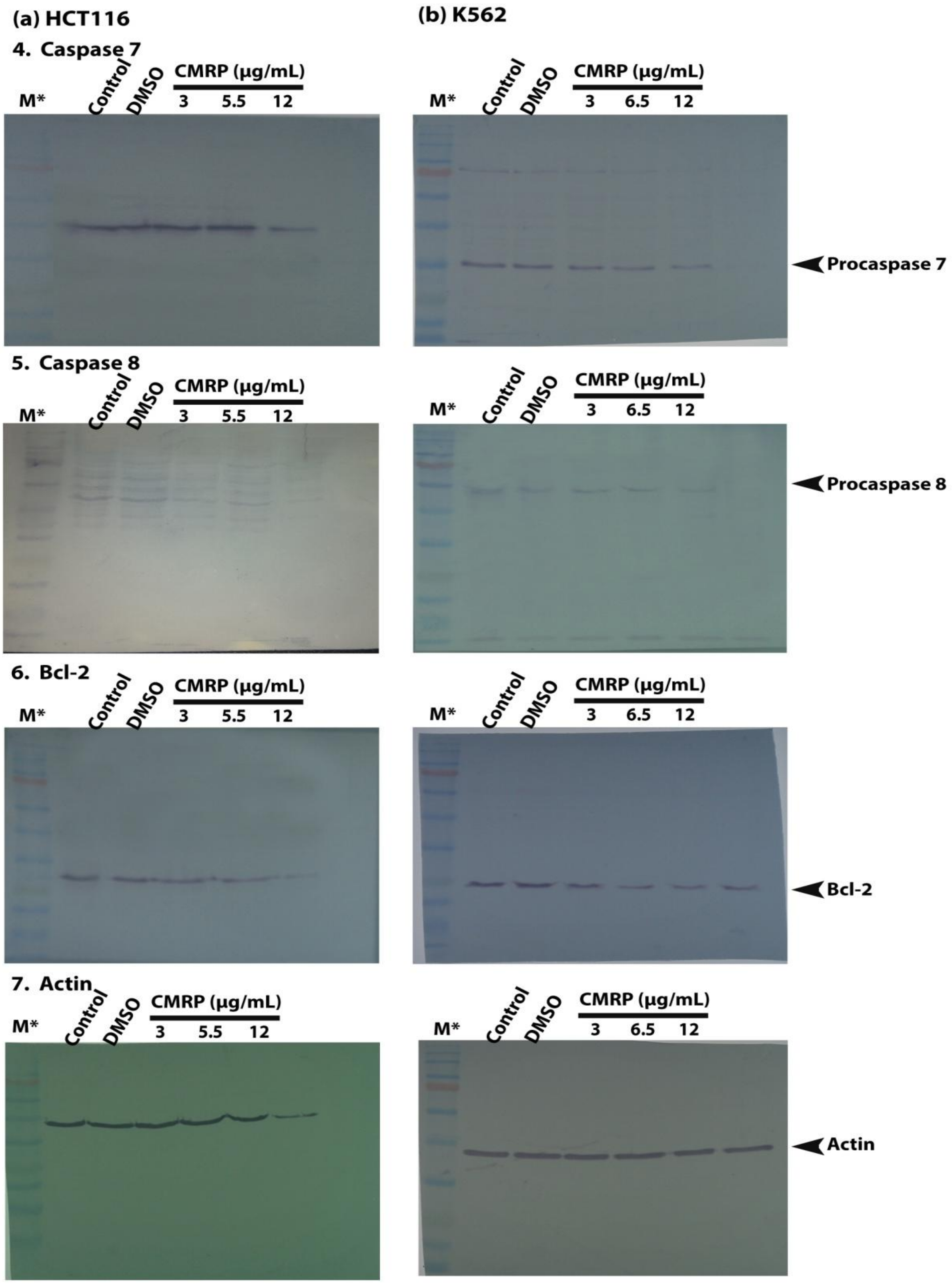

Supplementary Figure S10. Full length western blot presented in Figure 9 of the main article, Effect of CMRP on expression of apoptosis related proteins in HCT116 and K562 cancer cells analysed by western blotting using antibodies against caspase -7, -8, Bcl-2 and loading control actin. Lane M* represent prestained protein marker, with 12 prestained proteins covering a wide range molecular weights from 10 to 245 kDa, **(Figure continued in next page).**

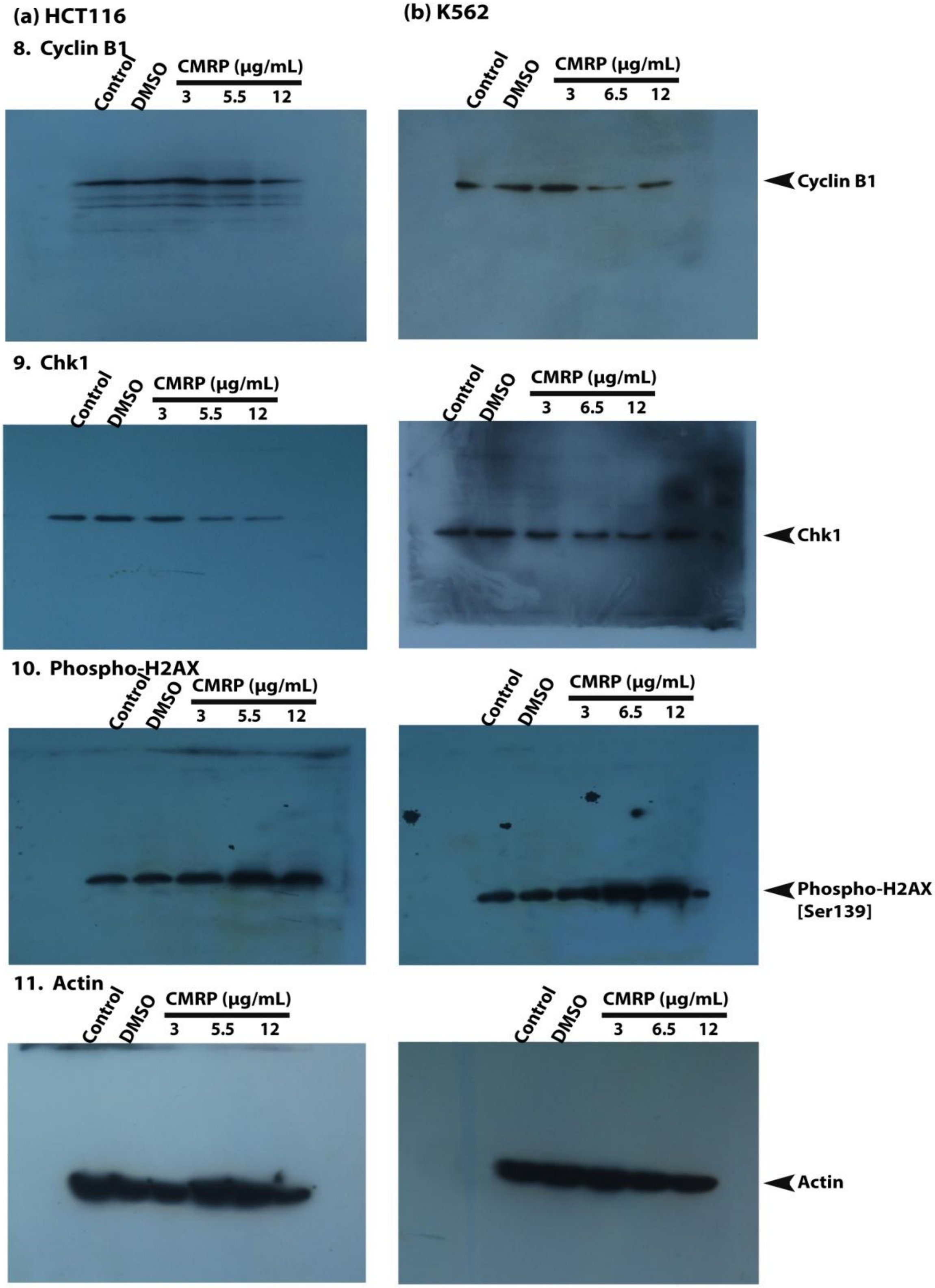

Supplementary Figure S10. Full length western blot presented in Figure 9 of the main article, Effect of CMRP on expression of apoptosis and cell cycle related proteins in HCT116 and K562 cancer cells analysed by western blotting using antibodies against cyclin B1, Chk1, phospho-H2AX and loading control actin. Lane M* represent prestained protein marker, with 12 prestained proteins covering a wide range molecular weights from 10 to 245 kDa.

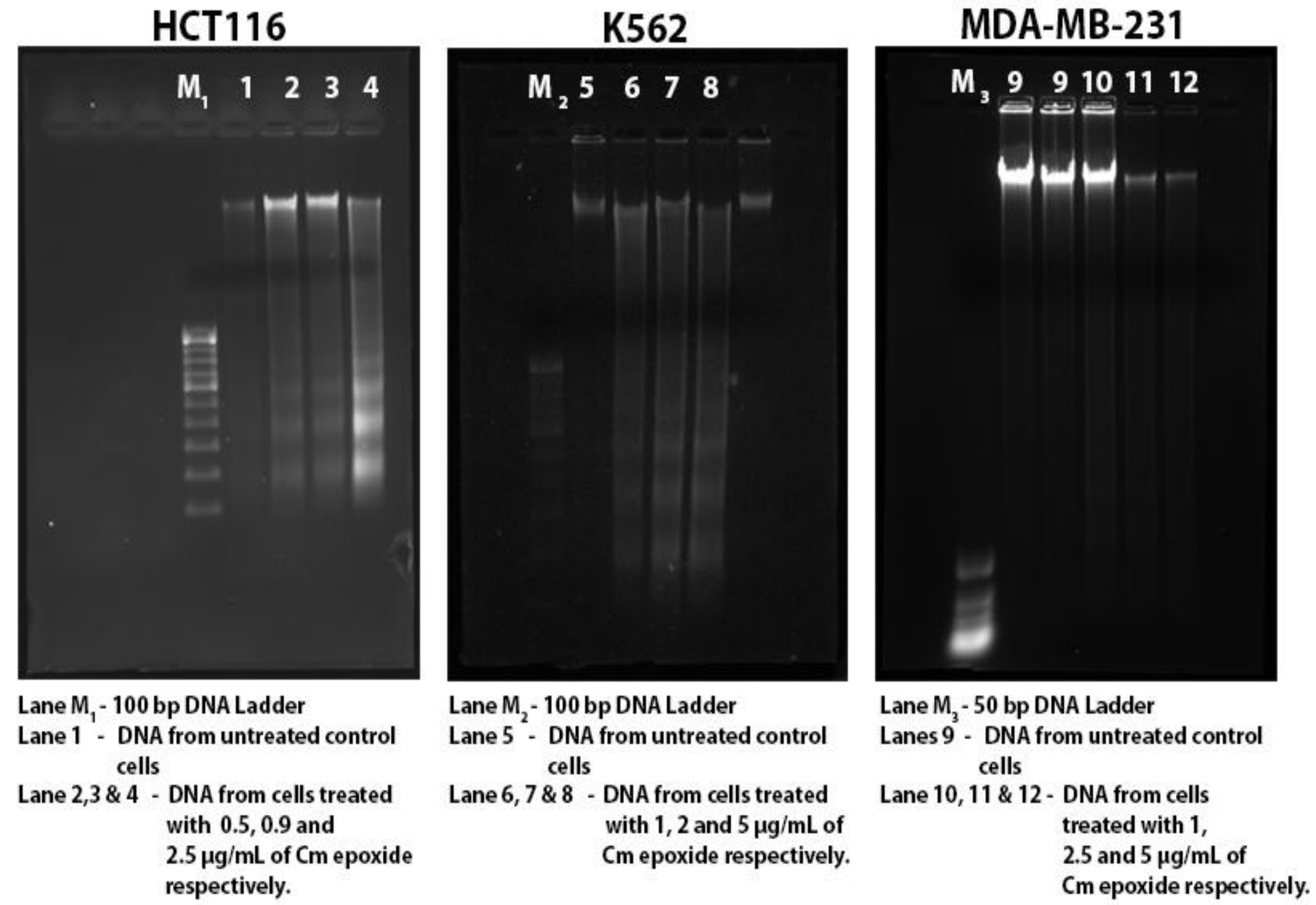

Supplementary Figure S11. Full length agarose gels displayed in Figure 10i of the main article, Cm epoxide-induced DNA fragmentation in HCT116, K562 and MDA-MB-231 cells.

.
